# FKBP Ligands—Where We Are and Where to Go?

**DOI:** 10.3389/fphar.2018.01425

**Published:** 2018-12-05

**Authors:** Jürgen M. Kolos, Andreas M. Voll, Michael Bauder, Felix Hausch

**Affiliations:** Department of Chemistry, Institute of Chemistry and Biochemistry, Darmstadt University of Technology, Darmstadt, Germany

**Keywords:** FKBP ligands, FKBP12, FKBP51, AIPL1, MIP, Rapamycin, FK506, SAFit

## Abstract

In recent years, many members of the FK506-binding protein (FKBP) family were increasingly linked to various diseases. The binding domain of FKBPs differs only in a few amino acid residues, but their biological roles are versatile. High-affinity ligands with selectivity between close homologs are scarce. This review will give an overview of the most prominent ligands developed for FKBPs and highlight a perspective for future developments. More precisely, human FKBPs and correlated diseases will be discussed as well as microbial FKBPs in the context of anti-bacterial and anti-fungal therapeutics. The last section gives insights into high-affinity ligands as chemical tools and dimerizers.

## Introduction

FK506-binding proteins (FKBPs) belong to the immunophilin family. Like other immunophilins many FKBPs possess a cis-trans peptidyl-prolyl isomerase (PPIase) activity. In addition, they can act as a co-receptor for the natural products FK506 and Rapamycin. By binding these drugs, a complex is formed, which exhibits immunosuppressive effects *via* a gain-of-function mechanism. Furthermore, the endogenous functions of FKBPs are increasingly being uncovered and several homologs have been linked to pathological processes.

**Figure 1:**
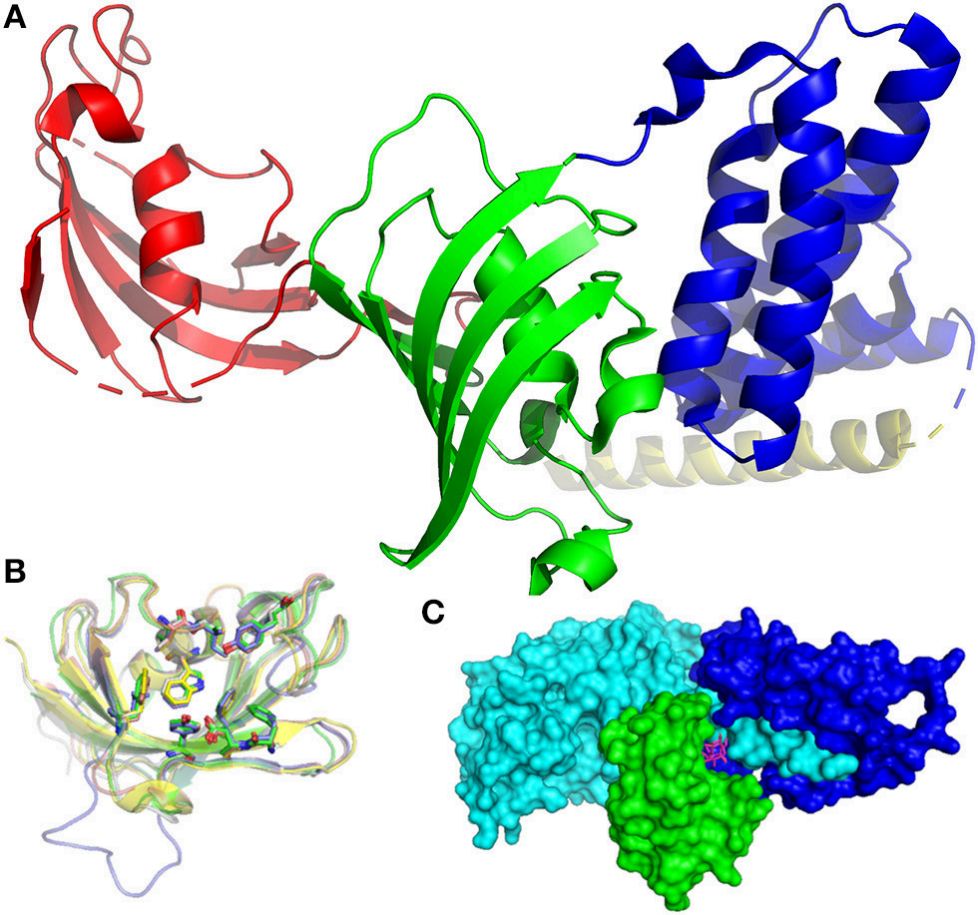
Structures of FKBPs and their interaction partners. **(A)** Structure of FKBP51 (pdb-ID: 1KT0). The FK1 domain is depicted in red, the FK2 domain in green, TPR domains in blue. The pale-yellow region corresponds to a putative calmodulin binding domain. **(B)** Superposition of the FK506-binding domains of FKBP12 (1FKJ, green), FKBP13 (4NNR, gray), FKBP25 (5D75, blue), FKB51 (3O5R, yellow), and FKBP52 (4LAX, salmon). The conserved active site residues are highlighted as sticks, the bound FK506 is omitted for clarity. **(C)** Inhibitory complex (1TC0) of FKBP12 (green), FK506 (pink sticks), and calcineurin (cyan and blue).

FKBPs are present in all eukaryotes, ranging from yeasts to humans, and expressed in most tissues. Mammalian FKBPs can be subdivided into four groups: the cytoplasmic, endoplasmic reticulum, nuclear, and TPR (tetratricopeptide repeats)-containing FKBPs. FKBP51, a member of the TPR-containing group, is depicted in Figure 1A. The FK506-binding domain (FK1) is shown in red. The tertiary structure of this domain is highly similar in most FKBPs which are therefore not easily distinguishable (Figure 1B). The crucial task for drug development is the exploitation of small variations in the biding pocket to achieve selectivity between different FKBPs.

This review will focus on the FK506-binding pocket of FKBPs and their ligands, including the prototypic natural products, synthetic analogs, endogenous ligands, and protein partners. Moreover, FKBP ligands will be discussed in the context of anti-microbials and as chemical tools.

## FKBP12 and FKBP12.6

FKBP12 was first described in 1989 by Harding et al. (1989) and Siekierka et al. (1989). With a molecular weight of 12 kDa, it is the smallest member of the FKBP family. It contains the PPIase core domain, which is found in many FKBPs. It occurs in most species and tissues and is essential for mammalian life. Knock-out of FKBP12 in mice produced an embryonic lethal phenotype due to severe heart defects attributed to interference with the ryanodine receptor (Shou et al., 1998). Furthermore, FKBP12 is linked to various diseases including Alzheimer's and Parkinson's disease, but its distinct role still needs to be elucidated.

The first ligands described for FKBP12 are the natural products Rapamycin and FK506. Both compounds are potent immunosuppressants in complex with FKBPs (best described for FKBP12) and act *via* a gain-of-function mechanism. The FKBP12-FK506 complex (depicted in Figure 1C) binds calcineurin (Griffith et al., 1995), a key enzyme in T-cell activation (Rosen and Schreiber, 1992; Kissinger et al., 1995), while the FKBP12-Rapamycin complex binds to the FKBP Rapamycin binding (FRB) domain of the mammalian target of Rapamycin (mTOR) (Liang et al., 1999; Banaszynski et al., 2005), a kinase involved in cell growth and cell proliferation (Waickman and Powell, 2012). Inhibition of both pathways leads to an immunosuppressive response. Therefore, FK506 and Rapamycin are used as drugs to stop allograft rejection in post-transplantation patients (Demetris et al., 1990; Fung et al., 1990; Todo et al., 1990; Armitage et al., 1991; Shapiro et al., 1991; Saunders et al., 2001; Zhang et al., 2018). Rapamycin is especially used in renal transplantation, where it displays less toxicity compared to related immunosuppressive agents (e.g., FK506) (Andoh et al., 1996) and in heart transplantations (Asleh et al., 2018). However, Rapamycin is often co-administered with cyclosporin A (CsA), since it was proven more active in combination with CsA or inactive on its own in some cases (Sharkey and Butcher, 1994; Patel et al., 2011). Although the immunosuppressive activity of FK506 is depending on the FK506-FKBP12 complex and calcineurin inhibition (Gold, 1997; Snyder et al., 1998), the neurotrophic activity is not. FK506 and other non-FKBP12-binding immunophilins displayed neuroprotective and neuroregenerative effects regardless of FKBP12-binding or FKBP12 presence at all (Winter et al., 2000; Costantini et al., 2001; Guo et al., 2001; Tanaka et al., 2002; Gold et al., 2005). Recently, it was shown that the neuritotrophic effects of FKBP ligands could be in part attributed to inhibition of FKBP51 (Gaali et al., 2015). Whether inhibition of FKBP12 can have beneficial neuronal effects is still unclear (Hausch, 2015). Therefore, high-quality FKBP12 ligands lacking immunosuppressive properties and off-target binding would be highly desirable.

### Nature-Inspired Ligands

**Figure 2:**
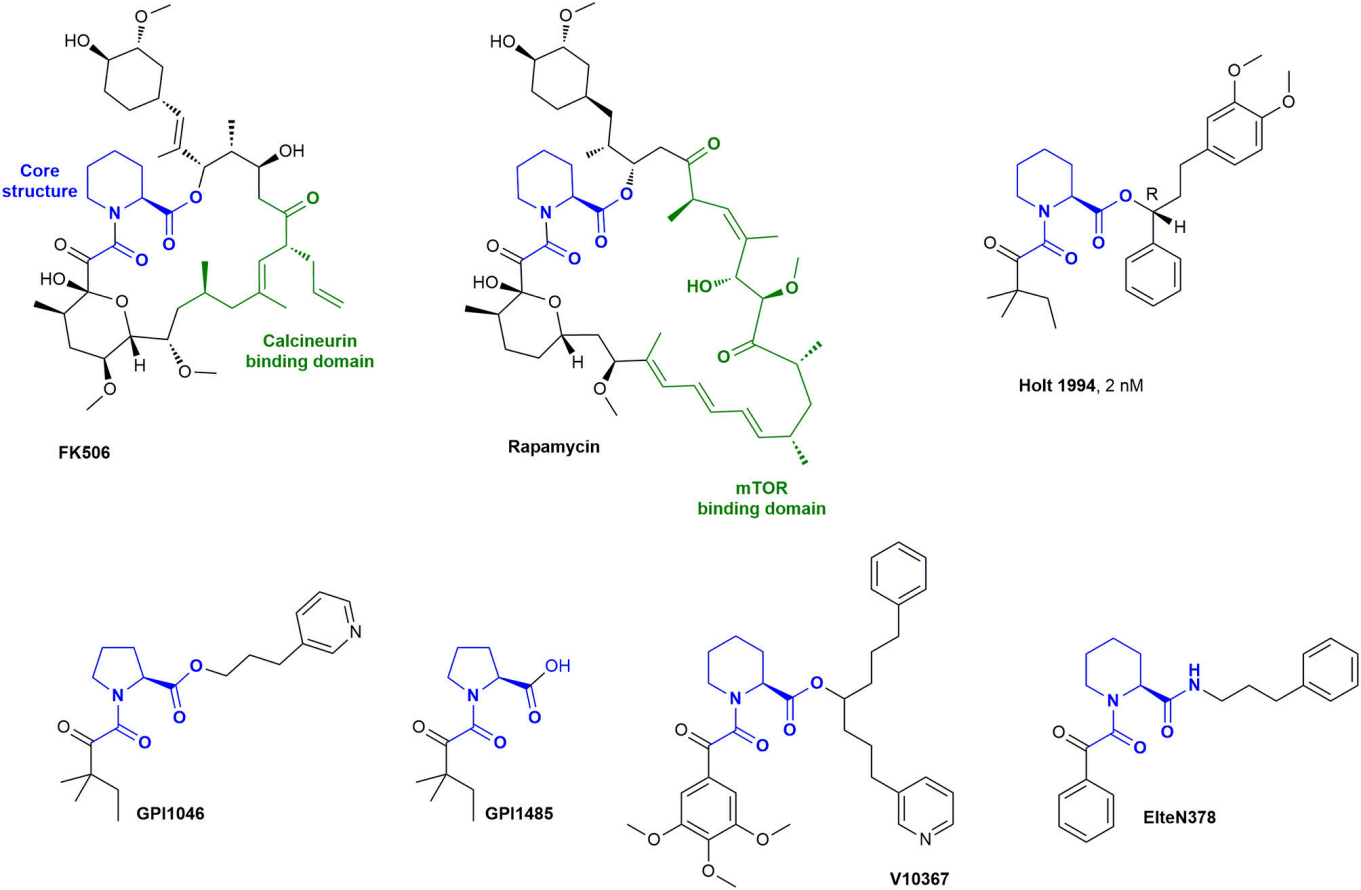
The natural drugs FK506 and Rapamycin and synthetic derivatives.

Crystal structures revealed that Rapamycin and FK506 bind to the pocket of FKBP12 *via* the pipecolate moiety, while the remaining part of the molecule points outwards and enables ternary complexes [Van Duyne et al., 1991a,b, 1993]. Early development of FKBP12 ligands focused on the improvement of these natural compounds by simplifying the structure and removing the loop which leads to immunosuppression. Various research groups developed synthetic cyclic and acyclic FK506 analogs by retaining the seemingly important pipecolinyl α-ketoamide function and modifying the outside loop (Figure 2) (Birkenshaw et al., 1994; Armistead et al., 1995; Luengo et al., 1995). The most potent inhibitor (2 nM) of this series had a t-pentyl residue on the diketo moiety and a diarylpropyl moiety on the ester. Notably, the stereochemistry highly affected the potency, since the R-isomer was ~40 times more active than the S-isomer (Holt et al., 1994).

Further variation of the side chains resulted in synthetic ligands, which were reported to bind with similar affinities to FKBP12 as FK506. Examples are GPI-1485 and the corresponding prodrug, GPI-1046 (Guilford Pharmaceuticals and Amgen) (Steiner et al., 1997), V10367 (Vertex Pharmaceuticals) (Armistead et al., 1995), and ElteN378 (Martina et al., 2013). GPI-1046 and GPI-1485 were discussed as a potential treatment option for Alzheimer's and Parkinson's disease (Steiner et al., 1997; Sauer et al., 1999; Ross et al., 2001; Marshall and Grosset, 2004; Caporello et al., 2006; NINDS NET-PD Investigators, 2007). However, numerous other research groups subsequently observed no or only negligible binding of GPI-1046 to FKBP12 (Harper et al., 1999; Winter et al., 2000; Bocquet et al., 2001; Eberling et al., 2002). GPI-1046 or GPI-1485 should not be regarded or used as FKBP ligands. Several research groups reported V10367 as a promising drug for neuroprotection and nerve regeneration (Costantini et al., 1998; Kupina et al., 2002; Klettner and Herdegen, 2003; Rosenstiel et al., 2003), but it is not known if the effect is derived from FKBP binding. ElteN378 was developed by Martina et al. and reported to have a K_i_ value of 3 nM determined by a fluorescent quenching assay (Martina et al., 2013).

### Polycyclic Ligands

**Figure 3:**
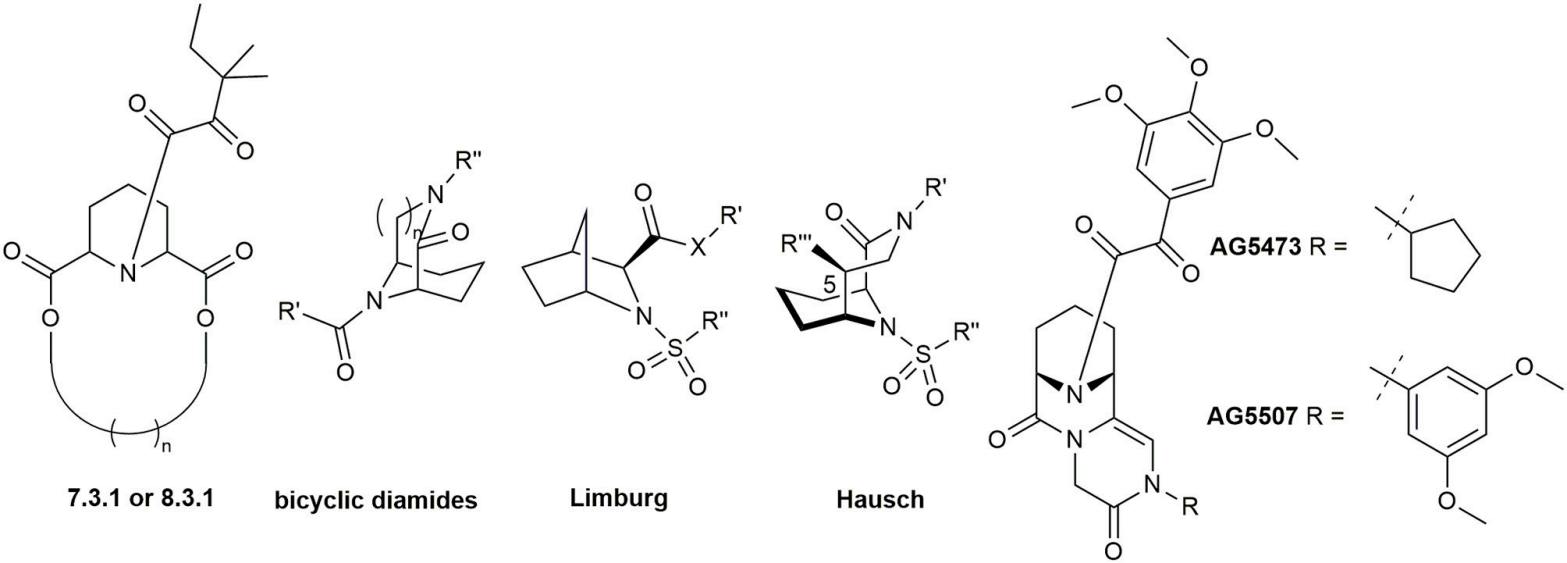
Bicyclic and polycyclic ligands for FKBPs.

By using computational modeling, Babine et al. discovered that bridged bicyclic structures fit well to the FKBP12 binding site. This was further confirmed with crystal structures (Babine et al., 1995) and led to the synthesis of many bicyclic scaffolds (Figure 3) including [7.3.1] and [8.3.1] macrocycles (Babine et al., 1996), bicyclic diamides (Dubowchik and Provencal, 2004), bicyclic aza-amides (Wang and Hausch, 2016), and chiral bicyclic proline analogs (Limburg et al., 2003). Katoh et al. developed polycyclic azaamides as inhibitors of isomerization activity (Katoh et al., 2003). Two compounds, AG5473 and AG5507, displayed high binding affinity for FKBP12, with 84 nM and 54 nm, respectively (Guo et al., 2000). Further derivatization of this scaffold by interconverting the α-keto-amide to sulfonamides, sulfamides, ureas and α,α-difluoro amides resulted in no or only marginal improvements in affinity (Hudack et al., 2006).

**Figure 4:**
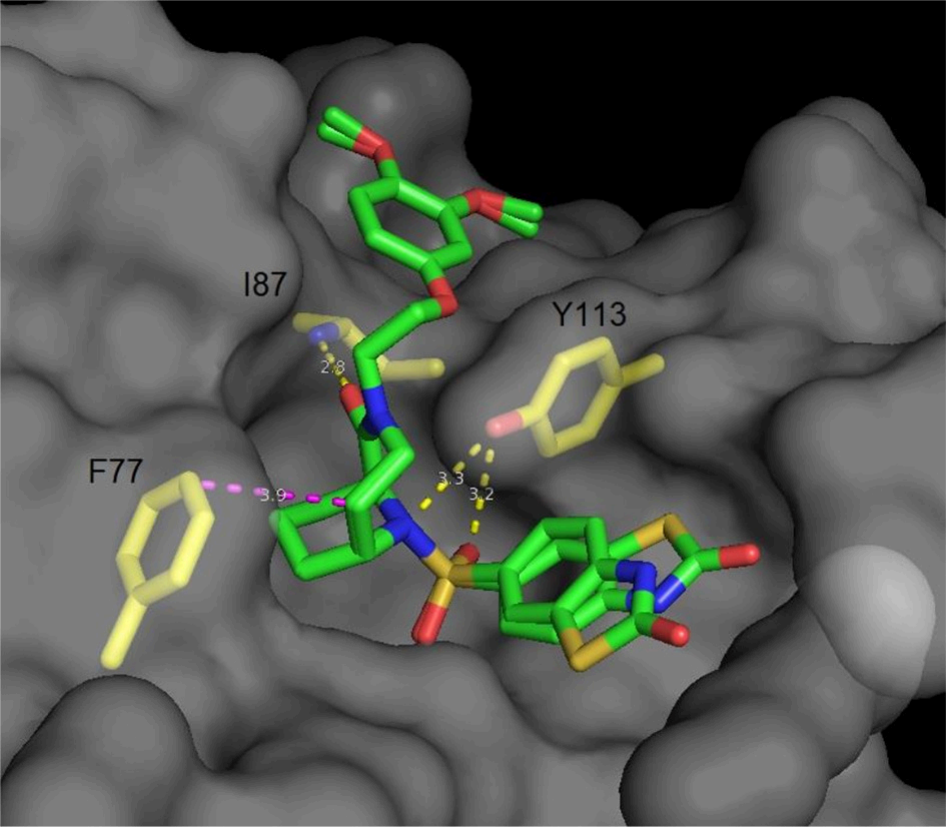
Cocrystal structure of a diazobicyclo [4.3.1]decan-2-one with the FK1 domain of FKBP51 (representative for FKBPs). The protein surface is depicted in gray; the key amino acids are highlighted as yellow sticks, while the ligand is green. Blue atoms correspond to nitrogen, red atoms to oxygen. Hydrogen bonds are shown in yellow, van der Waals interactions in magenta. The side chain of Lys^121^ has been omitted for clarity.

Following the idea of rigidification and bridging the pipecolate core, Hausch et al. developed a new class of 3,10-diazabicyclo[4.3.1]-decan-2-one sulfonamides. This very rigid structure closely mimics the active conformation of FK506, indicated by the dihedral O = C^2^-C^1^-N^3^ angle, which strongly resembles the FKBP-bound conformation of unconstrained FKBP ligands (both ~175°). Crystal structures elucidated the key interactions, some of which are the C^1^-O^1^ Ile^87^-N hydrogen bond (FKBP51 numbering), a bifurcated hydrogen bond of Tyr^113^ to one of the S = O oxygens as well as to N^10^ and van der Waals contacts of the installed linker with Phe^77^ (Figure 4) (Wang et al., 2013). Further improvements were achieved by modifications of the C^5^ position, resulting in highly ligand-efficient FKBP inhibitors with neurotrophic and anti-microbial activity (Bischoff et al., 2014; Pomplun et al., 2015, 2018). Although these ligands displayed high affinities toward FKBP12, it is noteworthy that several other FKBPs were also bound with nanomolar affinity and without selectivity between FKBP12 and its close homolog FKBP12.6.

### Biochemical Mechanisms of FKBP12 and FKBP12.6

The first described biochemical function of FKBP12 was the PPIase activity that is conserved in many, but not all FKBP homologs. So far, it has not been possible to unambiguously show how this catalytic activity affects cellular processes. Two signaling pathways are well established to be modulated by FKBP12, ryanodine receptors (RyRs) and transforming growth factor β (TGFβ)/activin-like receptors. However, both types of receptors are clearly regulated by FKBP12 through protein-protein interactions that utilize the PPIase active site but to the best of our knowledge do not involve a cis-trans isomerization step.

FKBP12 was repeatedly shown to bind to type I receptors of the TGFβ family. Specifically, it binds to the glycine- and serine-rich sequence (GS domain) of TGFβRI (Huse et al., 1999) or activin receptor-like kinase-2 (ALK2) (Chaikuad et al., 2012), stabilizing these kinases in an inactive conformation and blocking access of substrates. This is thought to prevent leaky signaling of the receptor kinases. Mutations in the GS region of ALK2 that abolish FKBP12 binding are associated with the connective tissue disease fibrodysplasia ossificans progressiva (FOP), but with milder symptoms compared to other ALK mutations (Machiya et al., 2018). FKBP12.6, the closest analog to FKBP12, with 85% amino acid identity and a highly similar tertiary structure (Snyder and Sabatini, 1995; Marks, 1996, 2002) is also able to bind to ALK2 (pdb-ID: 4C02) but its role, as well as those of other FKBP homologs, on TGFβ/activin signaling is unknown.

FKBP12 and FKBP12.6 are well documented components of RyR complexes, which intracellularly amplify Ca^2+^**-**signaling in myocytes to evoke muscle contraction. Recent cryogenic electron microscopy (Cryo-EM) structures have elucidated the FKBP binding site in the ryanodine receptor complex, where FKBP12 and FKBP12.6 occupied identical positions bridging the SPRY1 (a sequence repeat discovered in the splA kinase and ryanodine receptors) and the handle domains (Yan et al., 2014; des Georges et al., 2016). However, higher resolution structures are needed to elucidate the details of the FKBP-RyR binding interface and differences between FKBP12/12.6 and RyR1 and RyR2.

While the RyR1-FKBP12 complex is present in skeletal muscle, the homologous RyR2 and FKBP12.6 seem to have evolved as a specialized system to control cardiac muscle stimulation in mammals. Numerous studies have investigated the role of FKBP12.6 in heart biology, however, with highly controversial findings. Some reports suggest that FKBP12 is stronger associated with RyR1 while FKBP12.6 is stronger connected to RyR2 function (Li et al., 2012; Venturi et al., 2014). Others observed that there is no selectivity for RyR1 but specificity of cardiac RyR2 for FKBP12.6 over FKBP12 (Timerman et al., 1996) or at least higher affinity in heart cells toward FKBP12.6 (Guo et al., 2010; Oda et al., 2015). Fleischer et al. systematically investigated 18 amino acids, which differ between these two proteins. They identified three amino acids (Gln^31^, Asn^32^, and Phe^59^) which are responsible for selective binding of FKBP12.6 to RyR2. By site-directed mutagenesis they made triple mutants of FKBP12 and FKBP12.6 with the amino acids of the other isoform. The triple mutant of FKBP12 showed RyR2 selectivity, while the triple mutant of FKBP12.6 had no selectivity for RyR2. Control experiments displayed that both isoforms still bind to RyR1 (Xin et al., 1999). In an attempt to reconcile discrepancies regarding the role of FKBP12.6, Zhao et al. measured the stimulation of ryanodine receptors (primarily RyR2) by voltage-gated Ca^2+^ channels (primarily CaV_1.2_) in mouse cardiomyocytes (Zhao et al., 2017). They observed enhanced Ca^2+^ spark frequency and quicker voltage-gated Ca^2+^ channel-RyR2 coupling in FKBP12.6-deficient and FK506- or Rapamycin-treated cardiomyocytes.

Recently, FKBP12.6 was found to protect the heart from hypertrophy through inhibition of Ca^2+^/calmodulin mediated signaling pathways (Xiao et al., 2018). A pending question is how these observations translate to temporal RyR2-FKBP12.6 disruption in intact animals as well as to humans.

## FKBP13 and Other Endoplasmic FKBPS

The second characterized member of the FKBP family, FKBP13, shares 43% amino acid sequence identity with FKBP12 and has a conserved PPIase domain, which binds to FK506 and Rapamycin (Jin et al., 1991). In contrast to FKBP12, FKBP13 is a membrane-associated protein localized to the lumen of the endoplasmic reticulum (ER). It contains a C-terminal Arg-Thr-Glu-Leu (RTEL) sequence responsible for ER retention (Nigam et al., 1993; Bush et al., 1994). Further, FKBP13 is thought to play a role in protein folding in the ER (Walensky et al., 1998). Recently, FKBP13 was identified as a key regulator of protein homeostasis in long-lived plasma cells (Jeong et al., 2017). By interaction with immunoglobulin (Ig) the ubiquitination of Ig is facilitated, leading to lowered levels of ER stress. FKBP13 knockdown in cells increased the amount of secreted IgA, while overexpression resulted in decreased secretion of IgA. The latter effect was inhibited by treating cells with Rapamycin, possibly by blocking the binding site responsible for correct folding of IgA. Ishikawa et al. investigated the role of endoplasmic FKBPs as chaperones for collagen biosynthesis. Since collagen contains multiple proline residues, the cis-trans isomerization of the amino acid is thought to be the rate-determining step during triple-helix formation. Post-translational modifications of proline to hydroxyproline provide thermal stability, and therefore, they modulate the folding of the triple helix of collagen. FKBPs bind differently to these substrates. FKBP13 has a low affinity toward hydroxylated substrates, while FKBP19, FKBP22, and FKBP65 were found to prefer hydroxylated substrates (Ishikawa et al., 2017). However, since FKBP65 has four FKBP-like domains, which are poorly characterized regarding the binding of ligands, it is difficult to pin down the observed effects to one of these domains. FKBP65 is further associated with lysyl hydroxylase 2 (LH2), a key regulator of collagen cross-linking stability. The FKBP65-LH2-complex promotes dimerization of LH2 and thereby positively modulates its activity (Gjaltema et al., 2016; Chen et al., 2017). While FKBP65 knockout completely inhibits its PPIase activity and consequently LH2 mediated cross-linking, FK506 evokes only partial inhibition (~25%) (Zeng et al., 1998). More potent inhibitors could be used in LH2 associated pathologies, like fibrosis (van der Slot et al., 2005; Remst et al., 2013) and cancer metastasis (Eisinger-Mathason et al., 2013; Gilkes et al., 2013; Chen et al., 2015).

Although FKBP13 is mostly known for its function in the ER, it was also found in erythrocytes which lack ER. Walensky et al. reported the association of FKBP13 with the homolog 4.1G of the red blood cell protein 4.1 (4.1R) and suggested a function of FKBP13 in the membrane cytoskeleton of mammalian cells (Walensky et al., 1998). In the context of Alzheimer's disease, inhibition of FKBP13 (for instance by FK506 or Rapamycin) was found to destabilize presenilins, a family of transmembrane proteins, which are responsible for γ-secretase activity (Boulianne, 2006). Inhibition of the γ-secretase complex results in decreased formation of amyloid-β proteins, which are believed to play a key part in the development of Alzheimer's disease (Hamley, 2012).

## FKBP25

FKBP25 was originally purified from bovine tissue (Galat et al., 1992). It shows 97% homology with the human FKBP25 (hFKBP25) (Hung and Schreiber, 1992). The human FKBP25 is a nuclear protein which consists of two domains linked by a long random coiled loop (Prakash et al., 2015). While the C-terminal domain is a PPIase domain found in many FKBPs, the N-terminal basic tilted helix bundle (BTHB) domain is unique within the FKBP family (Helander et al., 2014). FKBP25 has been shown to interact with the histone deacetylases HDAC1 and HDAC2 as well as with their transcriptional regulator Yin Yang 1 (YY1) (Yang et al., 2001). Further, it interacts with DNA via both domains (Prakash et al., 2016b), with RNA (Dilworth et al., 2017) and with mouse double minute 2 (MDM2) (Ochocka et al., 2009). Recently, FKBP25 was identified to play a role in the proliferation of non-small cell type lung cancer cells (NSCLC) by modulating Sp1/HDAC2/p27 (Zhu et al., 2017). Furthermore, FKBP25 was found to be a microtubule-associated protein (MAP) that shuttles between nucleus and cytoplasm and stabilizes the microtubule network by direct binding of the C-terminal domain (Dilworth et al., 2018).

### FKBP Domain

The C-terminal domain binds immunophilin ligands like Rapamycin and FK506, although compared to other FKBPs the affinity for FK506 is substantially lower (Rapamycin Ki 0.9 nM, FK506 200 nM) (Jin et al., 1992; Liang et al., 1996; Prakash et al., 2016a). Most of the interactions are comparable, with the exception of Lys^170^, which forms 2 hydrogen bonds to Rapamycin. These bonds are not present in the crystal structure with FK506. Additionally, the interaction with Gly^169^ is lost, suggesting a lack of donor atoms responsible for the reduced affinity of FK506 (Prakash et al., 2016a). The crystal structures give an insight in the different binding to hFKBP25, but further experiments are necessary to evaluate the role of Lys^170^.

### BTHB Domain

Although no ligands were developed for the C-terminal domain of FKBP25, many research groups focused on the better understanding of the N-terminal part. This helix bundle domain was found to mediate protein-protein interaction for ubiquitination and therefore degradation of oncogene mouse double minute 2 (MDM2), which leads to an enhanced expression of tumor suppressor protein p53 (Ochocka et al., 2009). Recently, interaction with double-stranded RNA (dsRNA) (Dilworth et al., 2017) and a role in ischemia stress injury protection (Jiang et al., 2018) were disclosed.

### Full Length FKBP25

Prakash et al. determined the NMR solution structure of full-length human FKBP25 in order to investigate the DNA-binding mechanism of FKBP25. The C- and N-terminal domains of FKBP25 interact with each other; especially the α2 and α4 helices of the helix bundle domain and the basic loop of the FKBP domain play a role in binding to DNA. Furthermore, the basic loop was found to be larger compared to other FKBPs and rich in lysine residues, probably favoring DNA binding (Prakash et al., 2016b).

## AIPL1

The aryl hydrocarbon receptor-interacting protein-like 1 (AIPL1) belongs to the FKBP family because it harbors an FKBP-like domain. Interestingly, this domain is devoid of PPIase activity (Yadav and Artemyev, 2017). Mutations in the AIPL1-encoding gene were identified as one of the factors causing Leber congenital amaurosis (LCA), an autosomal recessive inherited retinopathy leading to blindness in early childhood. Compelling evidence has shown that AIPL1 functions as an obligatory chaperone for phosphodiesterase 6 (PDE6), a key protein that converts stimuli from photoactivated G proteins to electrical signals in retinal neurons (Gopalakrishna et al., 2016; Sacristan-Reviriego et al., 2017).

Importantly, the FKBP-like domain of AIPL1 was shown to bind farnesyl and geranyl moieties, which are essential posttranslational modifications of PDE6. Recently, the structural details of isoprenyl binding by the AIPL1-FKBP domain were elucidated (Yadav et al., 2017). X-ray and NMR-studies revealed that the core and loop-region of AIPL1-FKBP rearranges to open a hydrophobic cavity capable of farnesyl/geranylgeranyl binding.

## FKBP51 and FKBP52

### FKBP51

The FK506-binding protein 51 (FKBP51), encoded by the *FKBP5* gene, acts as a cochaperone for the heat shock protein 90 (Hsp90) (Riggs et al., 2003) and is best known for the negative regulation of glucocorticoid and progesterone receptors (Pratt and Toft, 1997; Cioffi et al., 2011; Schmidt et al., 2012; Dunyak and Gestwicki, 2016).

FKBP51 structurally consists of a N-terminal FK1 domain, which displays PPIase activity, a PPIase-like FK2 domain and a C-terminal TPR domain. The FK1 domain binds the immunosuppressive drugs Rapamycin and FK506 with slightly reduced affinities compared to FKBP12 (Rapamycin: *K*_i_ = 3.7 ± 0.9 nM, FK506: *K*_i_ = 104 ± 14 nM) (Kozany et al., 2009). It shares 51% amino acid identity with FKBP12 (Blackburn and Walkinshaw, 2011). The FK2 domain adopts a typical FKBP-like structure, but it neither shows measurable isomerase activity *in vitro*, nor does it bind FK506 or Rapamycin (Storer et al., 2011; Dunyak and Gestwicki, 2016). Interestingly, the FKBP51-FK2 domain has greater similarity to FKBP38 than its respective FK1 domain (Blackburn and Walkinshaw, 2011). The TPR domain forms the docking site for Hsp90 (Young et al., 1998; Assimon et al., 2015).

A study on the interaction of FKBP51 with the protein kinase B (Akt) showed that binding of FKBP51 to Akt is independent from FK506 binding pocket. Several inhibitors of the FKBP51-FK1 domain like FK506, FK1706 or Rapamycin were tested for their effect on Akt binding, but none of them displayed any mentionable results (Fabian et al., 2013).

Independent reports on the role of FKBP51 in the pathogenesis of psychiatric disorders and stress-related diseases reveal it to be an attractive drug target (Schmidt et al., 2012). In numerous studies FKBP51-deficient mice showed an improved stress resistance and an improved stress-coping behavior (O'Leary et al., 2011; Touma et al., 2011; Hartmann et al., 2012; Albu et al., 2014; Hoeijmakers et al., 2014; Sabbagh et al., 2014). A follow up study on stress resiliency in aged mice displayed a learning enhancement caused by the absence of FKBP51 (Sabbagh et al., 2014). In humans, genetic variations of the FKBP51-encoding gene *FKBP5* that were shown to enhance expression of FKBP51 (Klengel et al., 2013) are associated with various stress-related disorders, especially in an environmental background of early life trauma (Klengel et al., 2013; Zannas and Binder, 2014; Zannas et al., 2016). Promising studies on the involvement of FKBP51 in energy metabolism revealed an enhanced expression of FKBP51 in adipocytes and adipose tissue (Pereira et al., 2014; Sidibeh et al., 2018). Furthermore, FKBP51-knockout mice displayed a resistance to weight gain under both regular and high-fat diets and an improved glucose tolerance (Stechschulte et al., 2016; Balsevich et al., 2017). Recently, FKBP51-knockout mice showed lower hypersensitivity in different pain models, without affecting the detection of acute painful stimuli, which is an important protective mechanism (Maiarù et al., 2016, 2018). Notably, Maiaru et al. were also able to demonstrate that the pain regulatory role of FKBP51 can be dissociated from its regulation of mood.

Inspired by the well-established role of FKBP51 as a suppressor of the glucocorticoid receptor as well as its involvement in the nuclear factor “kappa-light-chain-enhancer” of activated B-cells (NF-κB) pathway, a research group from Boehringer Ingelheim recently explored the role of FKBP51 in models of glucocorticoid-resistant asthma (Kastle et al., 2018). Mice overexpressing FKBP51 in airway epithelial cells were resistant to low dose of glucocorticoids. Knockdown of FKBP51 also led to a 10-fold enhanced glucocorticoid sensitivity in A549 cells, suggesting FKBP51 as a target for glucocorticoid-resistant asthma. The mechanisms of FKBP51 interaction with the glucocorticoid receptor or NF-κB are still unclear. A pending question is, if FKBP51 inhibitors can evoke the same effect.

### FKBP52

FKBP52 is the closest homolog to FKBP51, sharing 70% sequence identity and an almost identical tertiary structure. It is encoded by the *FKBP4* gene and, like FKBP51, contains a N-terminal FK1 domain exhibiting PPIase activity, a FKBP-like domain (FK2) without isomerase activity and a tetratricopeptide repeat domain (TPR domain) located at the C-terminus (Young et al., 1998; Cheung-Flynn et al., 2005; Storer et al., 2011). The FK1 domain shares 53% amino acid identity with FKBP12 and also binds to Rapamycin and FK506 (Rapamycin: *K*_i_ = 4.2 ± 0.7 nM, FK506: *K*_i_ = 23 ± 3 nM) (Kozany et al., 2009; Blackburn and Walkinshaw, 2011). Antagonistic to FKBP51, FKBP52 acts as positive regulator of the glucocorticoid and progesterone receptors (PR) (Schülke et al., 2010; Gaali et al., 2015). One possible explanation for this antagonistic behavior may be the flexibility of a hinge, more exactly a short linker between the FK1 and the FK2 domain of FKBP52, and its modulation by phosphorylation (Bracher et al., 2013).

Mice lacking FKBP52 displayed female infertility and several defects in male sexual development, which is in line with the role of FKBP52 in hormone signaling, especially as an PR and androgen receptor (AR) regulator (Cheung-Flynn et al., 2005; Sivils et al., 2011; Guy et al., 2016). This hormone receptor activity makes FKBP52 an interesting target in hormone-driven tumors like prostate cancer, colorectal adenocarcinomas, breast cancer, and myelomas (Storer et al., 2011; Storer Samaniego et al., 2015). Recently, Joshi et al. were able to prove an interaction between FKBP52 and inhibitor of differentiation 4 (ID4) in castration-resistant prostate cancer (CRPR) *in vitro* (Joshi et al., 2017). In this type of cancer, prostate cancer cells adapt to the androgen-depleted environment of the prostate. ID4 is a dominant-negative helix-loop-helix protein that promotes *de novo* steroidogenesis and castration-resistance with a gene expression signature similar to constitutive AR activity in castrated mice. Their findings show, that ID4 regulates the AR activity by interaction with FKBP52, whereas in absence of ID4 FKBP52 potentiates AR signaling and leads to increased proliferation and tumor growth. Hence, selective inhibition of FKBP52 would be a valuable tool to further understand the molecular alterations underlying castration resistance in prostate cancers.

**Figure 5:**
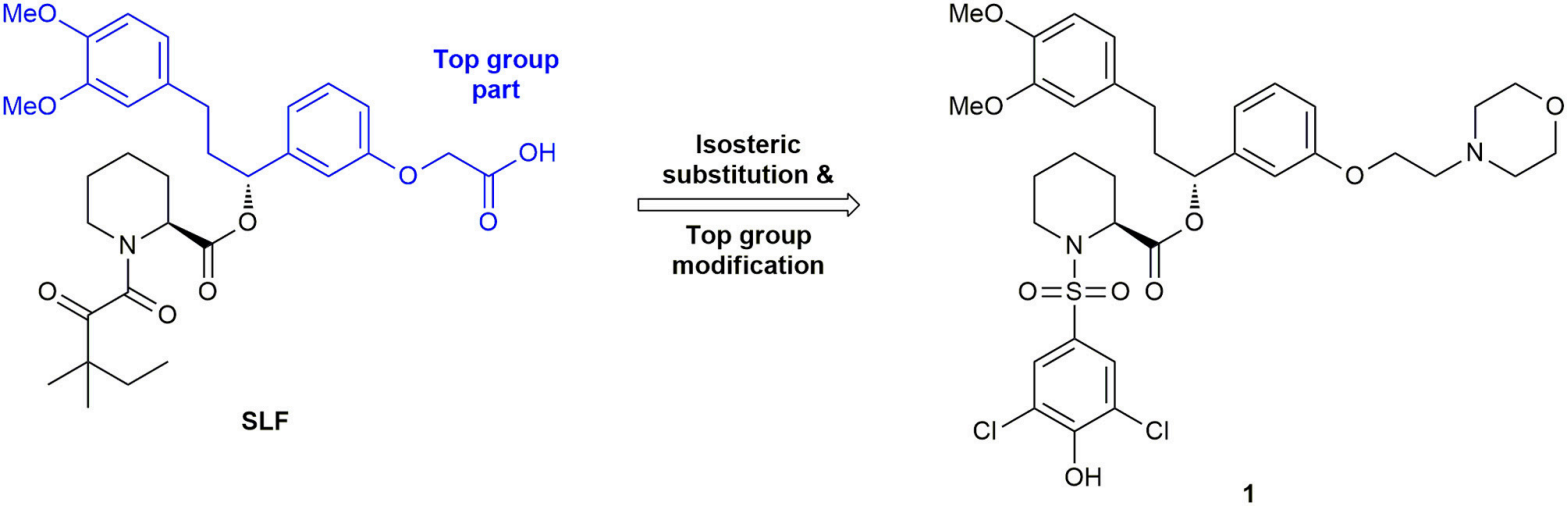
Development of early FKBP51 ligands.

The development of inhibitors for FKBP52 was initially strongly connected to that of FKBP51, since the FK506-binding pockets are highly conserved and show similar binding to almost all synthetically designed ligands except those of the SAFit (Selective Antagonist of FKBP51) class (Feng et al., 2015a). An early ligand for FKBP52 was an analog of SLF (Synthetic Ligand for FKBP, Figure 5), a ligand originally developed for FKBP12. The α-ketoamide is substituted by an aryl sulfonamide, bearing a chlorine in both meta-positions and a hydroxy substituent in para-position (*K*_i_ = 0.71 ± 0.1 μM) (Gopalakrishnan et al., 2012b). However, this ligand shows only marginal selectivity against FKBP51 or FKBP12.

To achieve selectivity for FKBP52 against FKBP51 and other FKBP's (especially FKBP12), it might be helpful to pick up on structure-based drug design at a point before selectivity for FKBP51 was obtained. Valuable data for the binding of FKBP52 to different ligands, as well as detailed analyses on the subtle differences in the binding domains of FKBP51 and FKBP52, are already existing (Bracher et al., 2013; Shi et al., 2018). In 2013 Bracher et al. suggested to exploit differences in the electrostatic potential of residue 85. In FKBP51 this residue is a Gln, while in FKBP52 it is a Glu. This offers the possibility to design suitable ligands for favored interactions with FKBP52 (Bracher et al., 2013).

Searching for FKBP52 modulators, the Cox group identified a molecule called MJC13, that specifically inhibits the FKBP52-enhanced AR activity in yeast and mammalian cells (De Leon et al., 2011). This compound was suggested to act by targeting the regulation of AR by FKBP52 through binding a regulatory surface on the AR and preventing the FKBP52-receptor complex from dissociation, not by selective binding to FKBP52 (Cox and Richer, 2017). The exact mechanism and binding mode of MJC is still unclear.

### Ligand Development

As for FKBP12, the natural products FK506 and Rapamycin were the first known potent inhibitors of FKBP51. FK506 was used to study the pharmacological role of FKBP51 in defined cellular models. Independent reports displayed a connection of FKBP51 to GR binding and the effects upon treatment with FK506 (Reynolds et al., 1999; Davies et al., 2005; Westberry et al., 2006). In 2013, März et al. showed that complexes of Rapamycin with larger FKBP homologs like FKBP51 can tightly bind to mTOR, thus forming ternary complexes that allosterically inhibit the kinase activity of mTOR. This effect was mainly discussed and interpreted in context of a ternary complex of Rapamycin and mTOR with FKBP12 (März et al., 2013). However, the primary effects of FK506 and Rapamycin are the inhibition of calcineurin (for FK506) and mTOR (for Rapamycin), which limits their use as a probe for FKBP51 in living organisms. The search for non-immunosuppressive immunophilin ligands for FKBP51 faces a selectivity issue between the homologs FKBP51 and FKBP52, since the FK506-binding sites in both proteins are completely conserved (Blackburn and Walkinshaw, 2011). Nevertheless, selectivity is crucial, due to the opposite effects on glucocorticoid signaling in cells and partially also *in vivo* (Feng et al., 2015a).

The first synthetic ligand for FKBP51 was compound SLF (Figure 5) with an affinity of 3.1 μM for FKBP51 (Holt et al., 1993; Keenan et al., 1998; Kozany et al., 2009). The process of ligand development was accelerated by structural biology and by the development of a medium-throughput assay for the FKBP ligands. In 2009, Kozany et al. used FL-SLF, a fluorescein derivative of SLF, for competitive fluorescence polarization assays for most FK506 binding-competent FKBPs (FKBP12, 12.6, 13, 51, and 52) (Kozany et al., 2009). Subsequently, this assay was used in a high throughput screening of more than 380,000 compounds, aiming to reveal binders to the FK506-binding pocket of FKBP51 (unpublished). However, no chemically tractable starting point other than FK506 or Rapamycin could be identified.

An efficient co-crystallization protocol yielding high-resolution structures of the FKBP51-FK1 domain has been established to support the development of inhibitors (Bracher et al., 2011). The crystal structures of FK506 and SLF in complex with the FK1 domain of FKBP51 served as chemical starting point for the structure-based rational design of potent and selective FKBP51 inhibitors.

In 2012, Gopalakrishnan et al. performed the first detailed structure-activity relationship analysis of small synthetic molecules for the human FKBPs 51 and 52 by systematically exploring the contributions of substituents on affinity (Gopalakrishnan et al., 2012a,b). Here, the pipecolic core turned out to be essential for the binding affinity. The tert-pentyl group in SLF was substituted by various hydroxyl-cyclohexyl moieties, supposed to mimic the pyranose in FK506 and Rapamycin. Unfortunately, these changes did not substantially increase the binding affinities of the inhibitors (Gopalakrishnan et al., 2012a). In another SAR study, the top group part of the molecule (see Figure 5) and the pipecolic core were retained, whereas the α-ketoamide was bioisosterically replaced by an aryl sulfonamide (Gopalakrishnan et al., 2012b). With a solid phase synthetic strategy, a small focused library was prepared, resulting in ligands with submicromolar affinities for FKBP51 and FKBP52. In addition, the top group was changed to a morpholine moiety, leading to ligand **1** (Figure 5) with 15 to 20-fold enhancement in affinity for both FKBPs compared to SLF (IC_50_ (FKBP51) = 456 nM, IC_50_ (FKBP52) = 710 nM, IC_50_ (FKBP12) = 115 nM) (Gopalakrishnan et al., 2012b).

**Figure 6:**
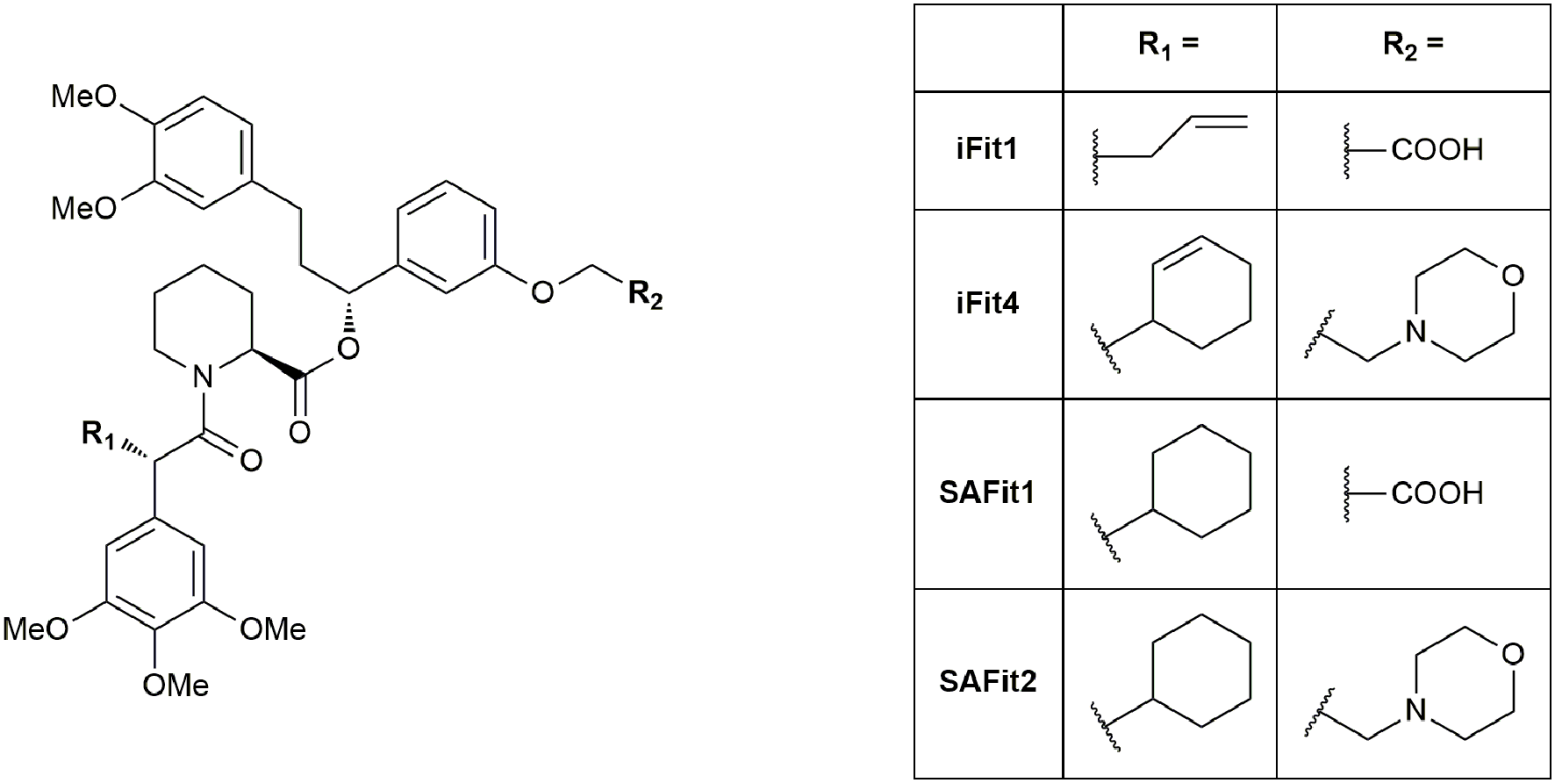
Ligands of the iFit/SAFit series.

To distinguish between FKBP51 and FKBP52 in a model system, Gaali et al. used a chemical genetics approach to generate artificial selectivity (Gaali et al., 2015). This approach was originally developed to selectively target FKBP12 fusion proteins (Clackson et al., 1998). Adapting this procedure, Gaali et al. generated FKBP51^F67V^ and FKBP52^F67V^ mutants, which featured enlarged FK506-binding sites and synthesized complementary ligands. The desired ligand potently bound to the mutated proteins FKBP51^F67V^ and FKBP52^F67V^, facilitating the desired study on neuronal differentiation in a cellular model. Interestingly, during the development, the researchers noticed that a structural analog (iFit1, Figure 6) retained some binding affinity to WT FKBP51 but not to WT FKBP52. Analysis of the crystal structure of the FKBP51-iFit1 complex revealed, that the α-carbon allyl substitution stabilized a conformational change compared to either the apo form or the FKBP51-FK506 complex by displacing Phe^67^. This conformational change is hard to achieve in FKBP52, since the resulting conformation is more strained due to the bulky FKBP52-specific residues Thr^58^, Trp^60^, and Val^129^. Synthesis of a small series with increased bulk of the α-carbon side chain revealed a cyclohexyl ring as the most potent residue for FKBP51 binding. The best ligands out of this series were termed SAFit ligands for Selective Antagonist of FKBP51 by induced fit, Figure 6). SAFit1 and SAFit2 have *K*_i_ values of 4 nM and 6 nM, respectively, and show over 10,000-fold selectivity for FKBP51 over FKBP52. Both, SAFit1 and SAFit2, do not show any immunosuppressive activity, since they lack the effector domain responsible for binding to calcineurin or mTOR. Although these are the best FKBP51 ligands published so far, further optimizations are needed to improve the physicochemical properties, which strongly deviate from the properties required for CNS-directed drugs (Wager et al., 2010). In particular, SAFit1 and SAFit2 display too high molecular weights (748 and 803 g/mol, compared to 426 g/mol for 90% of CNS drugs) and too low ligand efficiencies (LE = 0.21 and 0.19, compared to 0.37 for 90% of CNS drugs) (Gaali et al., 2016).

**Figure 7:**
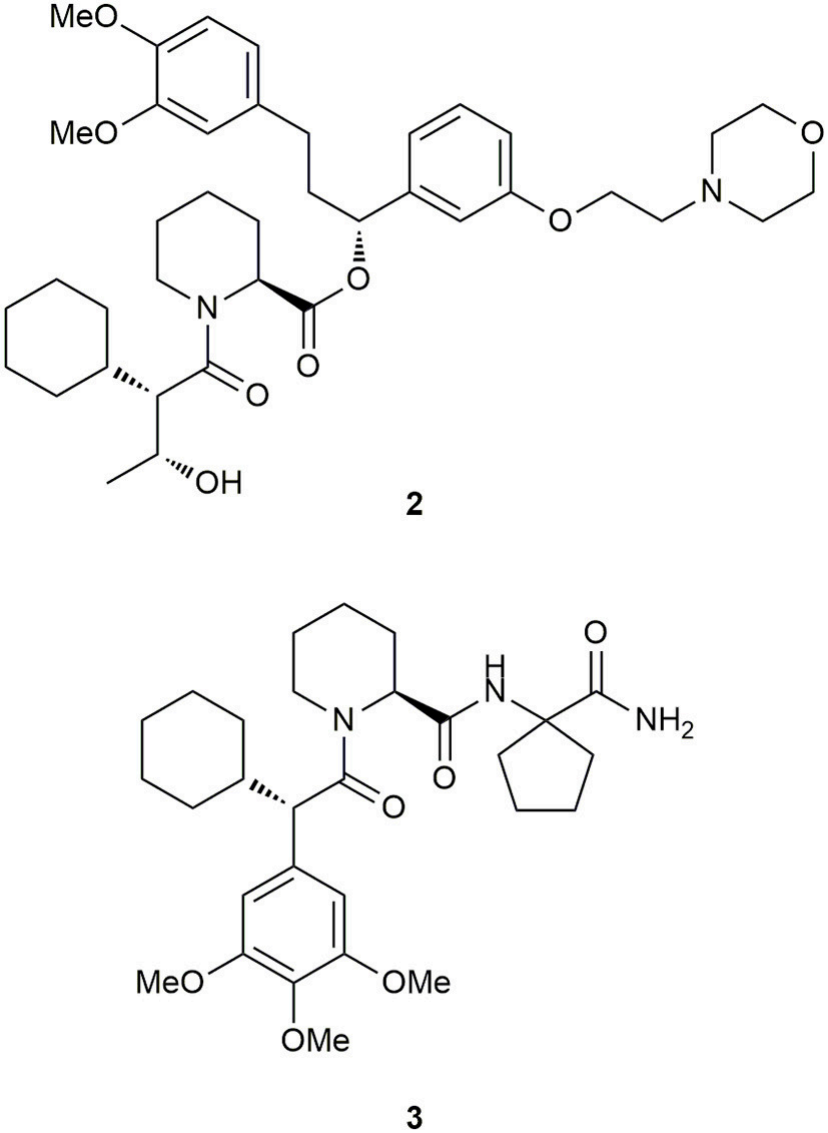
Derivatives of SAFit.

Based on the breakthrough with the SAFit ligands, Feng et al. investigated the requirements for binding to the newly discovered conformation to gain further insights into the mechanism (Feng et al., 2015b). Since the cyclohexyl ring was found to be the most important structural feature for selectivity, the researchers explored the importance of the neighboring trimethoxyaryl substituent of the SAFit ligands. They gradually removed the methoxy substituents leading to a 30–100-fold loss in binding affinity for FKBP51. Further reduction of the phenyl ring revealed, that at least an additional methyl group in the α-position seems necessary to retain selectivity for FKBP51 over FKBP52. Subsequently, the authors applied a Crimmins-type aldol reaction to introduce short carbon chains bearing a hydroxy function at C^10^ with defined stereochemistry. Out of this series, ligand **2** (Figure 7) displayed the best affinity toward FKBP51 (*K*_i_ = 700 nM) and confirmed the FKBP51-selective induced-fit binding mode, which seems not to be influenced by the substitution pattern at C^10^. Another optimization approach by Gaali et al. focused on the substitution of the top group part of the SAFit ligands by using a solid-phase synthesis strategy (Gaali et al., 2016). Replacement of the potentially labile pipecolic ester group by a series of amides, resulted in **3** (Figure 7), containing a geminally substituted cyclopentyl ring and displaying 20-fold selectivity against FKBP12 (FKBP51: *K*_i_ = 0.14 ± 0.02 μM, FKBP12: *K*_i_ = 3.8 ± 0.006 μM) and no appreciable binding to the antagonistic FKBP52. Notably, the molecular weight of **3** was reduced by 34% (MW = 529.7 g/mol, compared to SAFit2) and the ligand efficiency (LE = 0.25) was improved.

Recently, Shi et al. computationally analyzed the molecular binding mechanism and the binding trajectory of iFit4 (Figure 6) to FKBP51 and FKBP52 (Shi et al., 2018). They used molecular dynamics simulations, binding free energy calculations and unbinding pathway analysis to gain insights into the isoform selectivity. After conformational stability evaluation, they suggested that the preference of FKBP51 and FKBP52 for the “Phe^67^-in” and “Phe^67^-out” states are different, and therefore, the most important factor for the selectivity. This difference is structurally exploited by the cyclohexenyl ring in iFit4. A further potential site for structural optimization is Gln^85^ in FKBP51 (Glu^85^ in FKBP52), as suggested previously (Bracher et al., 2013).

LeMaster et al. analyzed the structural distribution of the residues of the FK1 domain of unliganded and iFit-bound FKBP51 by NMR studies (LeMaster and Hernandez, 2015). As a result, they proposed conformational selection as the mechanism responsible for the binding to the iFit ligands, in contrast to the induced fit mechanism proposed by Gaali et al. They suggested that a closely related conformational transition to that of the iFit-bound FK1 domain is also populated in the unliganded FK1 domain. It should be noted that the binding trajectory of SAFit ligands most likely involves conformational selection and induced fit mechanism. The mechanistic details when and how Phe^67^ is flipped out remain to be elucidated.

### Pharmacological Studies with SAFit2

With the SAFit ligands in hand, it was possible to unambiguously distinguish between FKBP51 and FKBP52. These ligands serve as probes for FKBP51 interactions in cellular and animal models. While SAFit1 is the ligand of choice in cellular experiments (due to better solubility and slightly better off-target profile), SAFit2 has much better pharmacokinetic properties and is the current gold standard for *in vivo* studies (Romano et al., 2015). Using SAFit2, Hartmann et al. were able to show reduced anxiety-related behavior in mice (Hartmann et al., 2015).

The research group of Géranton investigated the potential of FKBP51 as a pharmacological target for the treatment of persistent pain. Utilizing the selective FKBP51-inhibition through SAFit2, the research group was able to reduce mechanical hypersensitivity seen in joint inflammatory and neuropathic pain states in female and male mice (Maiarù et al., 2016, 2018). Furthermore, the selective FKBP51-inhibition also reduced the hypersensitivity in a translational model of chemotherapy induced pain.

Balsevich et al. demonstrated that chronic treatment with SAFit2 displayed the same metabolic effects in mice as a FKBP51 knockout, specifically body weight reduction and glucose tolerance improvement (Balsevich et al., 2017). The authors identified a novel association between FKBP51 and AS160, a substrate of AKT2, which is involved in the glucose uptake. This interaction was reduced in samples from SAFit2-treated mice through disruption of the AS160-FKBP51 complex. Consequently, the authors proposed FKBP51 as a mediator between stress and the development of type 2 diabetes.

Recent research on glioma showed, that inhibition of FKBP51 decreased the PD-L1 levels (Programmed Death Ligand 1) in glioma cells, suggesting FKBP51 as a potential target of immunomodulatory strategies for glioblastoma treatment (D'Arrigo et al., 2017). Romano et al. found an interaction of FKBP51 with the subunits of the IκB kinases (IKK) in melanoma cells Interestingly, the IKK-FKBP51 interaction does not seem to be dependent on the PPIase activity of FKBP51, but the kinase activity of IKK is. The IκB kinase complex is one part of the NF-κB signal transduction cascade and the activation of NF-κB is a major factor of melanoma resistance to apoptosis (Romano et al., 2015).

### Different Ways of FKBP51 Inhibition

Assimon et al. showed that FKBP51 and FKBP52 have a strong preference for binding to Hsp90 over Hsp70 (Assimon et al., 2015). Recently, Kumar et al. illuminated the binding mode of the Hsp90 C-terminal peptide to the TPR domain of FKBP51 using a combination of x-ray crystallography, differential scanning fluorimetry and molecular dynamics simulation (Kumar et al., 2017). They found, that the TPR domain of FKBP51 mainly interacts with the C^90^ peptide via the highly conserved amino acids Lys^272^, Glu^273^, Lys^352^, Asn^322^, and Lys^329^. The binding takes place via a di-carboxylate clamp, which is a common mode of action for TPR domain-containing proteins. Ligands targeting the TPR domain were suggested as an alternative approach for inhibition of FKBP51 function. However, it will require highly negatively charged compounds that have to compete with an intracellular protein-protein interaction (for depression in the brain). It remains to be seen, whether the binding requirements of the TPR domain can align with the pharmacokinetic properties necessary for drug-like FKBP inhibition.

Another approach to target the molecular function of FKBP51 was recently described by Sabbagh et al. (2018). In a high-throughput assay, they identified benztropine mesylate as a ligand, which is able to attenuate the suppression of GR activity in an FKBP51-dependant manner. Since benztropine does not inhibit the PPIase activity of FKBP51, the underlying mode of action is different to the aforementioned ligands like SAFit. Further mechanistic studies are necessary to better evaluate the potential of this promising approach.

## Microbial FKBPS

**Figure 8:**
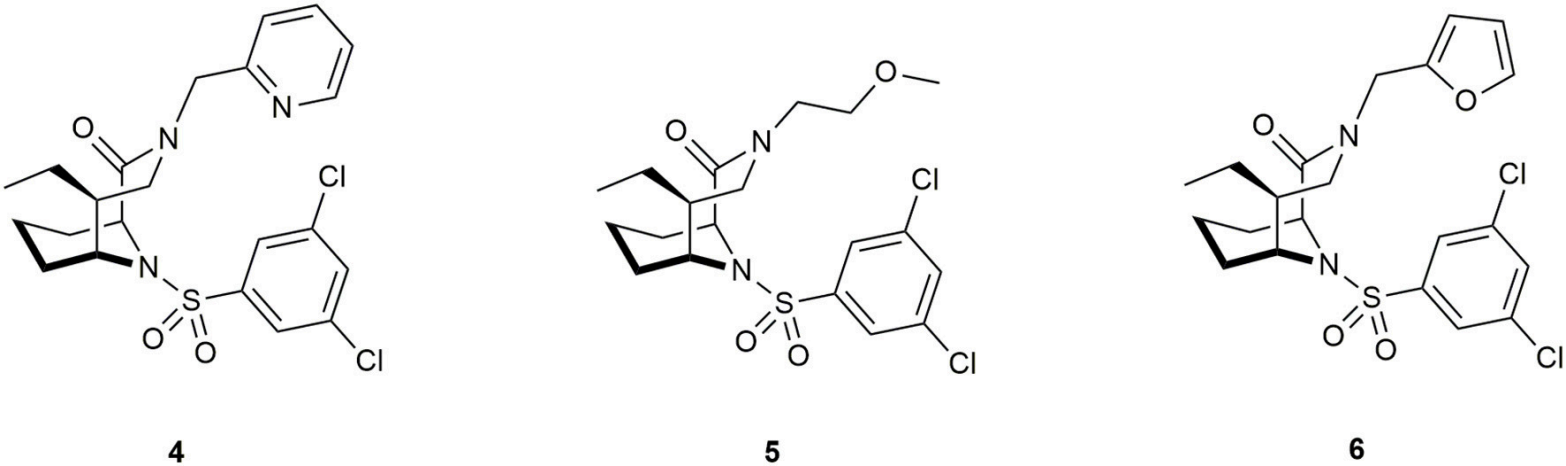
Ligands for the pathogens *Plasmodium falciparum, Legionella pneumophila, Chlamydia pneumoniae*, and *C*. *trachomatis*.

Antibiotics that target essential pathways in bacteria are subject to strong natural selection, which evokes a rapid rise of resistance (Lynch and Wiener-Kronish, 2008). Newer approaches focus on nonessential virulence factors like bacterial secretion or quorum sensing as targets (Baron, 2010; Sintim et al., 2010). One important virulence factor in several gram-negative bacteria is a close homolog to human FKBP12 called macrophage infectivity potentiator (MIP). This was first described in *Legionella pneumophila*, the causative agent for Legionnaires' disease. It potentiates the replication rates in human phagocytic cells and is therefore required for intracellular infection (Cianciotto et al., 1989). MIP and MIP-like PPIases are widely distributed in gram-negative bacteria (Ünal and Steinert, 2014), making this family a precious target for drug development. Structure determination of the MIP protein from *L. pneumophila* by crystallization with FK506 (Riboldi-Tunnicliffe et al., 2001) and by NMR spectroscopy in a complex with Rapamycin (Ceymann et al., 2008) revealed that both drugs bind *via* the pipecolate moiety to the PPIase domain of MIP, indicating a strong similarity between these complexes compared to the FKBP12 complexes with the respective ligand. Therefore, the pipecolate functioned as a starting point for ligand development. Juli et al. built upon work of Holt et al. (1993, 1994) and synthesized novel derivatives of this scaffold. Although one compound displayed high activity against the FKBP domain of *Legionella* MIP (IC_50_ = 6 μM), no influence on the replication of *L. pneumophila* in human cells was observed (Juli et al., 2011). Subsequent improved analogs, however, inhibited replication of *Chlamydia trachomatis* and reduced intracellular survival of *Neisseria* species (Reimer et al., 2016; Seufert et al., 2016). Recently, Pomplun et al. developed bicyclic ligands with high affinities for microbial FKBPs and MIPs (Ligands **4**, **5**, and **6**, Figure 8), including *Plasmodium falciparum, Legionella pneumophila* as well as *Chlamydia pneumoniae* and *trachomatis*.

The best ligands were able to inhibit proliferation of *L. pneumophila* and reduce infectivity of aforementioned *Chlamydias*. and decrease parasitemia in *P. falciparum*. However, the mode of action still needs to be elucidated. The authors suggested a key role of the PPIase domain and raised the question of participation of human FKBPs (Pomplun et al., 2018). Rasch et al. synthesized novel non-pipecolate ligands by combining two previously described FKBP-binding motifs, namely cycloheximide (Christner et al., 1999) and adamantane (Babine et al., 1995; Norville et al., 2011). These derivatives displayed lower IC_50_ values and improved selectivity for MIP (although FKBP12 was still 10-fold better inhibited) and inhibited bacterial growth in liquid culture. Notably, the effect was independent of MIP, since the same effect was shown in Δ*mip*-mutants (Rasch et al., 2015).

Recently, interest arose in L-685,818 (a competitive antagonist of FK506), which binds to FKBP12 but displays no immunosuppressive effects and does not inhibit calcineurin (Dumont et al., 1992; Organ et al., 1993). Nambu et al. were interested in the development of new treatments for systemic fungal infections, especially the two important pathogenic fungi, *Cryptococcus neoformans* and *Aspergillus fumigatus*. The growth of these fungi is dependent on calcineurin and can therefore be inhibited by FK506, as was shown before (Odom et al., 1997a,b; Cruz et al., 2000; Steinbach et al., 2006, 2007; Lev et al., 2012). Since FK506 also exhibits effects e.g. immunosuppressive activity and calcineurin inhibition in mammalian cells, it is essential to prevent these cells from interactions by blocking the binding site of FKBP12. Therefore, Nambu et al. developed antagonists for FK506, which were designed to preferentially permeate mammalian cells, but not fungal pathogens. The best compounds of this study bound as tightly to FKBP12 as FK506 and were able to inhibit *A. fumigatus* proliferation up to 24% (compared to untreated controls), when co-administered with FK506 (Nambu et al., 2017). Further improvements regarding the selectivity of permeation as well as studies in mice are needed before this concept can be used in antifungal therapy.

## Chemical Tools/Chemically Induced Dimerization Utilizing FKBPS

**Figure 9:**
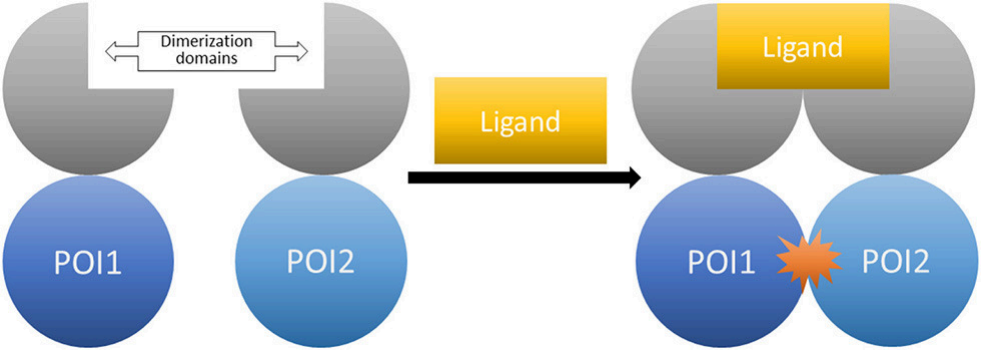
The CID system (here shown for homo-dimerization). Fusion proteins of the proteins of interest tagged with the respective dimerization domains. The controlled interaction starts upon administration of the dimerizer ligand.

The general idea for chemical dimerization with FKBP ligands is based on constructing fusion proteins consisting of a dimerization-domain and proteins of interest (POI). The aim is to induce the interaction of these proteins by addition of a small molecule. Afterwards, the follow-up cellular responses can be observed (Figure 9).

Several of the natural immunophilin ligands were found to be useful as chemical tools in the field of chemically induced dimerization (CID). For CIDs utilizing FKBPs, a fusion protein of the POIs and the immunophilin is necessary. An example for a natural asymmetric dimerizer is Rapamycin and its analogs (rapalogs), which bind FKBP12 and the FRB domain of mTOR1 (mammalian target of Rapamycin complex 1). Another example is FK506, which binds FKBP12 and calcineurin. Symmetrical dimerization can be induced by treatment with a bivalent ligand like synthetic dimers of FK506 or Rapamycin. This dimerization process is mostly used to observe cellular processes, some of which are the localization of signal proteins, the activation of receptors and the regulation of DNA transcription. As there are already several reviews on CIDs (Kley, 2004; Gaali et al., 2011; Stanton et al., 2018), this review will only give a short overview of earlier publications and focus on developments over the last years.

### Natural Immunophilin Ligands

**Figure 10:**
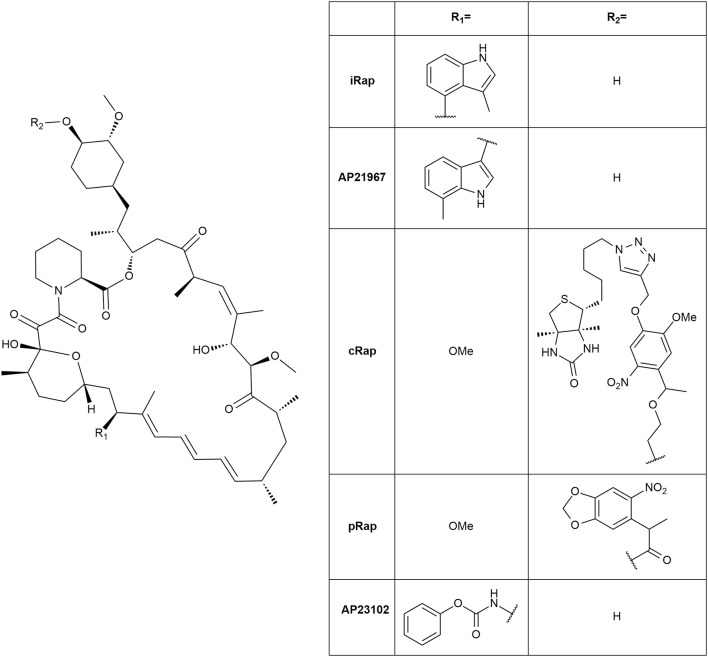
Structures of rapalogs.

The natural immunophilin ligands FK506 and Rapamycin are well established as CIDs with Rapamycin as the most prominent agent, possibly due to the easy handling of the FRB domain (Rivera et al., 1996; Ye et al., 1999; Kohler and Bertozzi, 2003; Komatsu et al., 2010; Robinson et al., 2010). The immunosuppressive properties of Rapamycin and the unspecific binding of endogenous FRB and FKBP12 can confound the actually intended results. Thus, many non-immunosuppressive Rapamycin derivatives and engineered cognate protein mutants were created using the “bump-and-hole” approach. These derivatives abolished binding to the wild type FRB and selectively bound to a FRB triple mutant (T2098L, W2101F, and K2095P) instead (Liberles et al., 1997; Stankunas et al., 2003). Further developments on the FRB binding loop improved the pharmacokinetic parameters of the rapalogs for instance AP21967 (Pollock et al., 2002) and iRap (Figure 10) (Inoue et al., 2005). Additionally, photoinducible variants were designed including cRap (Umeda et al., 2011) and pRap (Karginov et al., 2011).

FK506 was derivatized resulting in a bumped version (C^40^-p-phenoxyphenyl-FK506) and a cognate calcineurin mutant (Clemons et al., 2002). Additionally, a symmetric bivalent dimerizer of FK506 (FK1012) was synthesized, which abolished binding to calcineurin (Spencer et al., 1993). Recently, Guo et al. simplified the synthesis of this dimerizer by a one-step thiol-ene reaction (Guo et al., 2014).

### Fully Synthetic Bivalent Dimerizers

**Figure 11:**
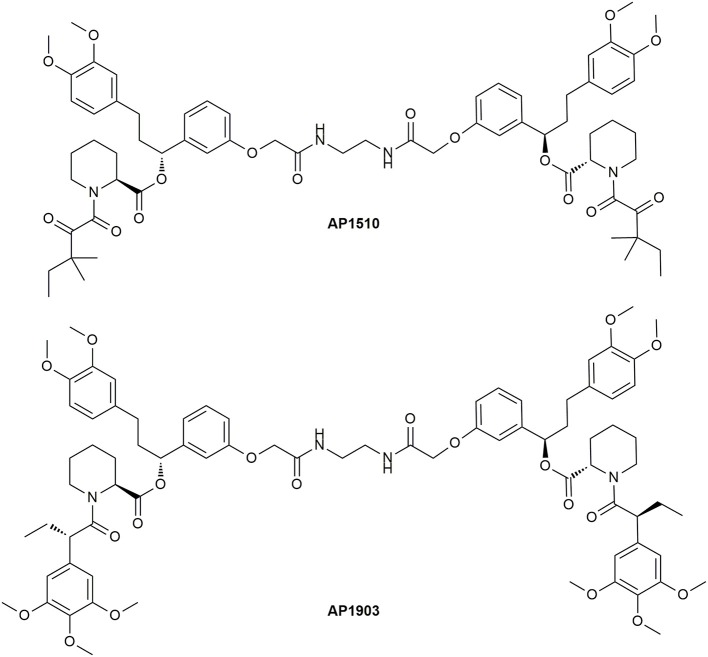
Structures of the bivalent ligands AP1510 and AP1903.

Fully synthetic but unselective FKBP ligands were developed by Holt et al. (1993). Further derivatization at the propyl bridged biphenyl moiety yielded an improved ligand regarding the affinity toward FKBP12. This monomeric ligand is mostly known as SLF (Figure 5). The bivalent version synthesized by Ariad Pharmaceuticals was named AP1510 (Figure 11) (Amara et al., 1997; Keenan et al., 1998).

**Figure 12:**
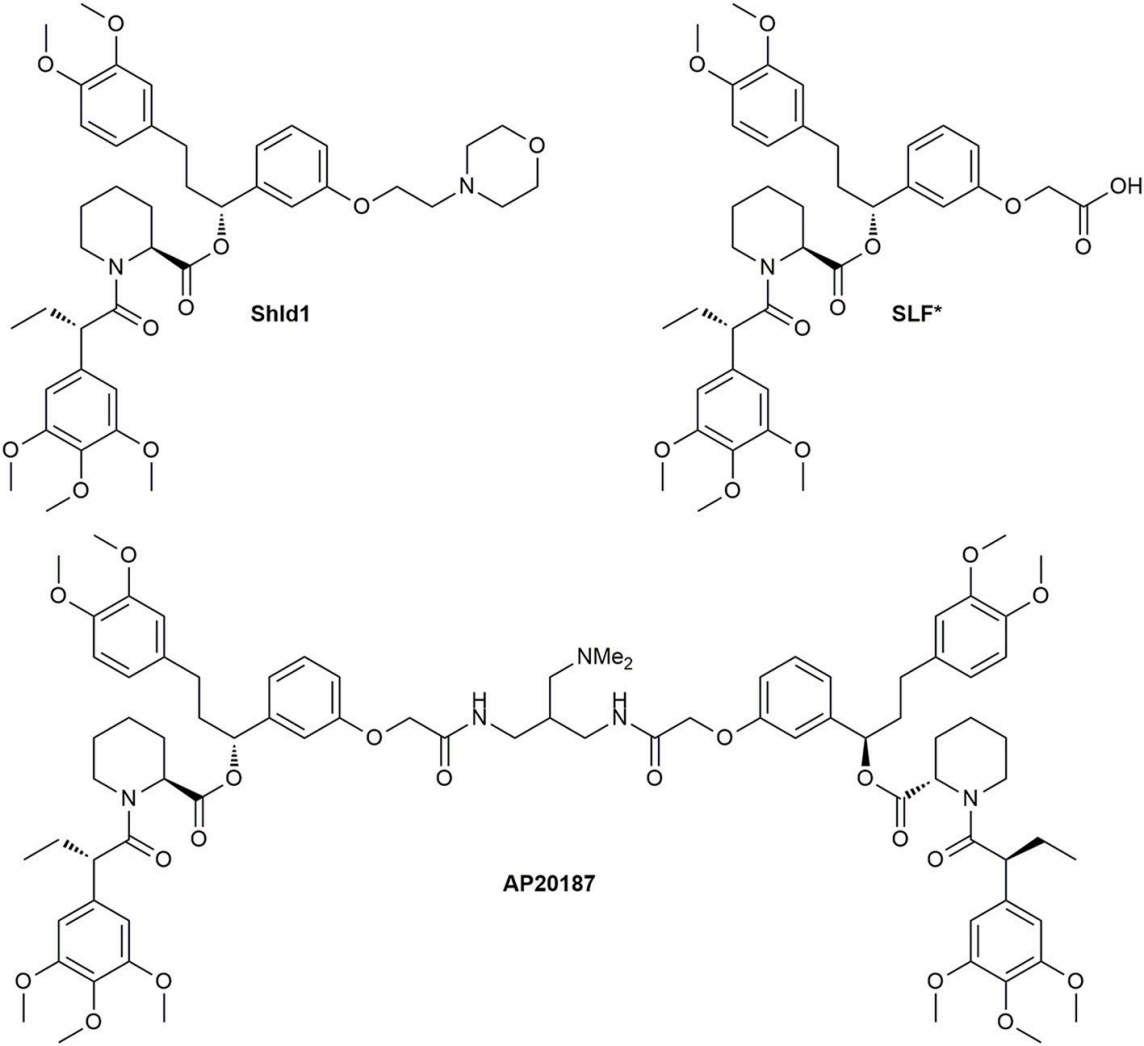
Structures of the selective bivalent dimerizer AP20187 and its monomeric precursors.

The undesirable binding of endogenous FKBPs was abolished by a “bump-and-hole” approach. Clackson et al. developed the selective bivalent ligand AP1903 for the FKBP12^F36V^ mutant (Figure 11) (Clackson et al., 1998). Regarding the nature of the linker for the symmetric CIDs a variety of derivatives were synthesized, but the most utilized one is AP20187 (Figure 12), which is nowadays sold by TaKaRa Bio. The superiority of this ligand is based on its increased solubility in aqueous solution. Yang et al. further developed monomeric forms (Yang et al., 2000), which were improved by Banaszynski et al. and named SLF^*^ and Shld1 (Figure 12) (Banaszynski et al., 2006).

Interestingly, the Shld1 synthesis was recently optimized by Jorgensen and Bols (2018).

### Usage of FKBP Ligand CIDs as Chemical Tools

CIDs are used in hetero/homodimerization and oligomerization, especially for the activation of receptors, some of which are the TGF-β receptor (Stockwell and Schreiber, 1998), epithelial growth factor receptor, hepatocyte growth factor and thrombopoietin receptor (Li et al., 2002), membrane tyrosine kinases (Spencer et al., 1993), insulin receptor (platelet-derived growth factor β) (Yang et al., 1998) and chimeric insulin receptor LFv2IRE (Cotugno et al., 2004). Furthermore, CIDs can be used to induce activation of apoptosis (Belshaw et al., 1996; Amara et al., 1997; Tsuruma et al., 2004; Landskroner-Eiger et al., 2010). For instance, an inducible mutant of caspase-9, iCasp9, which binds AP20187 was engineered to initiate apoptosis *in vivo*. Mice, transfected with colorectal cancer stem cells containing iCasp9 in their genome, were found to have strongly decreased tumor sizes after treatment with AP20187 (Kemper et al., 2012). The directed and controlled cell death is also used as a suicide switch in the study of transfected and modified T cells *in vivo*. Accordingly, caspase-8 was hybridized to be able to activate apoptosis utilizing AP20187 and therefore preventing unwanted proliferation (Khaleghi et al., 2012). T cells used in tumor therapy were modified to use iCasp9 and AP1903 (Clackson et al., 1998; Iuliucci et al., 2001) in the same manner by Zhou and Brenner (2016). RapaCas9, a caspase-9 dimerizable by Rapamycin treatment, was constructed recently to utilize the readily available drug (Stavrou et al., 2018).

**Figure 13:**
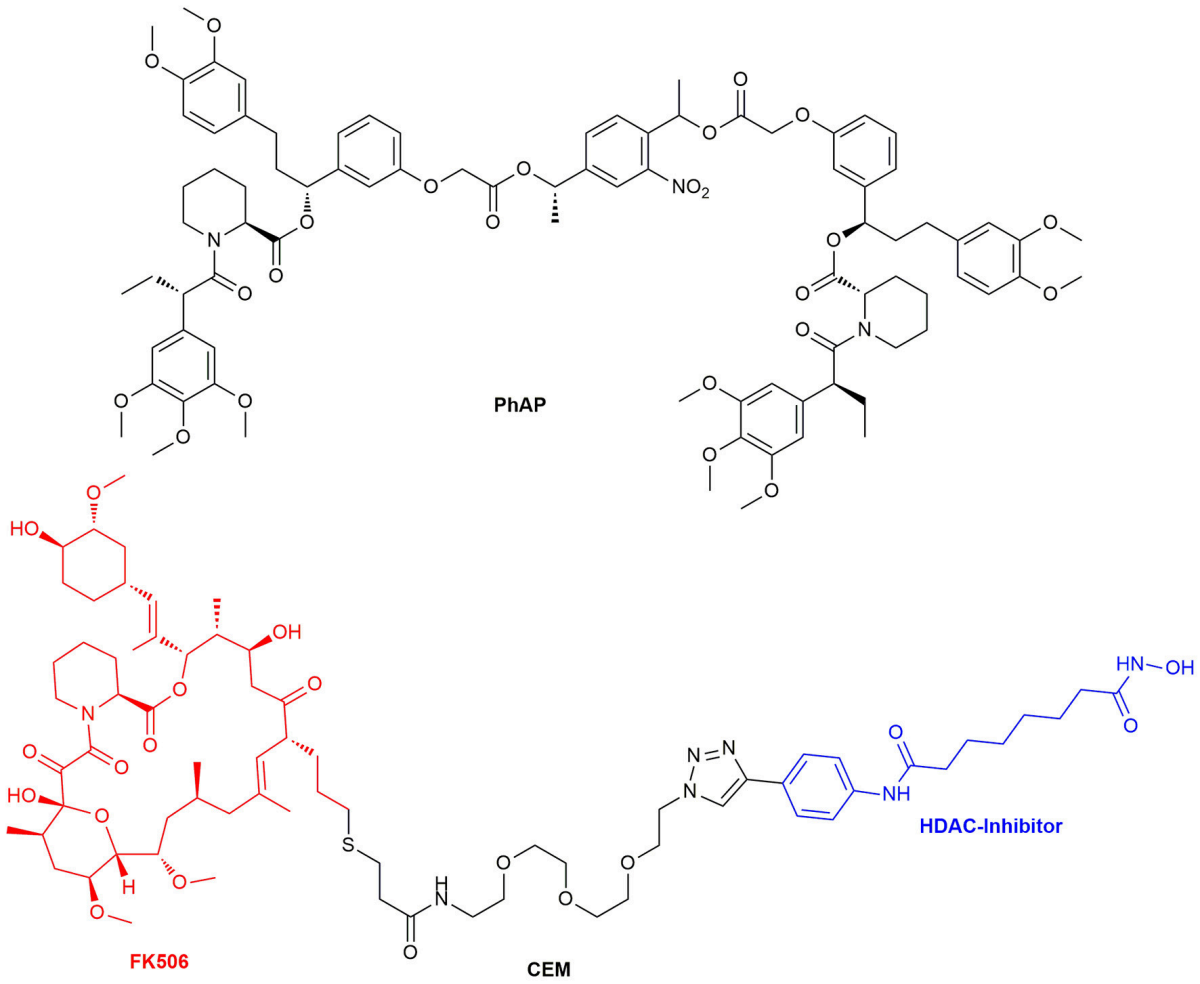
Structure of the chemical epigenetic modifier consisting of a FK506 and HDAC-Inhibitor moiety and the structure of the photocleavable selective bivalent dimerizer PhAP.

In 2014 Ahmed et al. introduced a photocleavable variant of AP20187 (PhAP) (Figure 13), which enables reversible dimerization. This was tested in cells by an endosome dispersion assay employing the fusion protein EGFP-FKBP-LC8_TRAP_. EGFP (enhanced green fluorescent protein) was fused to the FKBP12^F36V^ mutant and LC8_TRAP_. LC8_TRAP_ is a dynein light chain (LC8) low affinity binding peptide, which binds tightly to LC8 after dimerization. Interference with the dynein promoted processes leads to a visible change in endosome dispersion. PhAP was found to behave in a similar manner as AP20187, although it is possible to reverse the induced effect by the dimerizer with an additional handle (Ahmed et al., 2014). Proteins and receptor complexes, which not only need dimerization but oligomerization for activation, could be introduced by a system developed by Inobe et al. By adjusting the configuration and linker regions of fusion proteins of FRB and FKBP12, they were able to reach controlled Rapamycin inducible tetramer formation which was verified by tetramerizing aminopeptidase N and p53 (Inobe and Nukina, 2016). Recently, Chen et al. researched on the insulin receptor pathway and were able to differentiate between the insulin receptor types of the insulin-like growth factor receptor 1 (IGF1R) and the insulin receptor (InsR) as well as the respective hybrid of the heterodimer of both (HybR). The bivalent homodimerizer AP20187 and the heterodimerizer AP21967 were used as CIDs (Chen et al., 2018).

Another field where CIDs are applied is the regulation of transcription. Analogously to the yeast-two-hybrid (Y2H) assay, Rivera and Ho used CIDs to affect DNA transcription utilizing the gal-4 and zinc finger homeodomain 1 (ZFHD1) promoter regions (Ho et al., 1996; Rivera et al., 1996). A similarly developed method, the yeast-three-hybrid assay (Y3H), is mostly used to scan for small molecule protein targets or in a screening for specific protein mutations (Licitra and Liu, 1996; Althoff and Cornish, 2002; Kley, 2004). New methods for bacterial genome control were introduced by Becker et al. They designed homodimeric DNA looping proteins (TALEs), which are able to repress DNA transcription at a certain point by adding the dimerizer AP20187 (Becker et al., 2018). This system is comparable to the yeast-two-hybrid system for chemical transcription regulation. More recently, researchers focused on DNA modifications and epigenetics. Konishi et al. showed, that the telomeric repeat binding factor 2 (TRF2) binds to core histones to protect chromosome ends from DNA damage response and thus loss of telomeric DNA. This was achieved by dimerizing fusion proteins of a core histone with TRF2 with the rapalog AP21967 (Konishi et al., 2016). Furthermore, DNA methylation and its further impact were investigated by engineering a ten-eleven translocation protein (TET). TET2, which catalyzes the transformation of 5-methylcytosine (5mC) to 5-hydroxymethyl cytosine (5hmC) on DNA strands, plays a role in the first steps of DNA demethylation. TET2 was genetically split and each part fused to FKBP12 to control epigenetic remodeling by addition of CIDs (Lee et al., 2017). To further understand the link between epigenetic markers and gene expression, a CID-inducible FIRE-Cas9 system was established. Here, a Cas9 and a chromatin regulating enzyme complex [Hp1/Suv39h1 or mSWI/SNF (BAF)] can be dimerized with histones using Rapamycin. This showed that recruitment of these complexes modifies nearby histone marks (Braun et al., 2017).

Another recently published tool for the repression of DNA transcription utilizes histones. As a certain histone acetylation state is repressing transcription, Butler et al. developed a chemical epigenetic modifier (CEM, Figure 13) consisting of FK506 and a HDAC enzyme inhibitor which amplifies the restrictive acetylation state. Similar to the Y2H assay, Gal4 and FKBP12 were fused together to be able to recruit HDAC upon CEM administration to a specified gene region with Gal4 as promoter. HDAC itself consists of a huge protein complex and inhibition of one site does not inhibit its activity. The HDAC complexes affected local histone marks as reporter gene expression decreased by nearly 50% (Butler et al., 2018). In addition to the CRISPR/Cas9 system for genome editing a split Cas9 architecture for inducible genome editing was created. This inducible Cas9 might enable tissue specific activation of gene transcription with orthogonal dimerizer strategies in the future (Zetsche et al., 2015).

**Figure 14:**
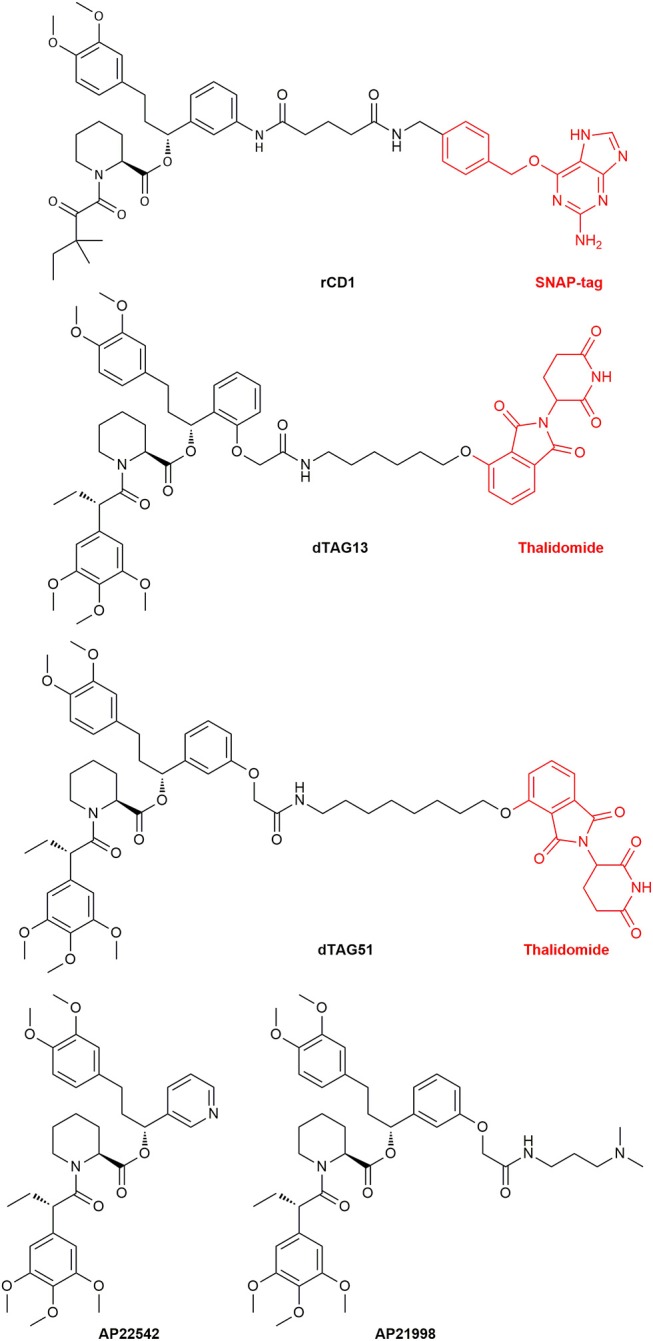
The structure of the reversible chemical dimerizer rCD1, the structures of the PROTACs dTAG13 (ortho Shld1 regioisomer) and dTAG51 and the structures of AP22542 and AP21998 which are used in reverse dimerization to dissolve the FKBP12 dimer.

Another major research area where CIDs are useful is the elucidation of the mechanisms of protein functions and the transport and localization of proteins and vesicles. The latter can be achieved by fixating signaling proteins on to the cellular membrane and switching on cellular processes. This was done with the guanine nucleotide exchange factor son of sevenless (SOS) (Holsinger et al., 1995). Furthermore, nuclear trafficking control (Xu et al., 2010) and transport proteins like dynein, kinesins and myosins were investigated (Kapitein et al., 2010). To study lipid signaling of phosphatidylinositol 3,4,5-trisphosphate (PIP3) release, a reversible dimerizer consisting of a SNAP-tag site and the Shld ligand was synthesized. This reversible chemical dimerizer (rCD1) (Figure 14) has a SNAP-tag for permanent binding, but the Shld ligand can be competitively eluted with FK506 or the non-immunosuppressive rapalog AP23102.

To observe lipid signaling a Lck-ECFP-SNAP fusion protein was constructed. The Lck protein localizes the construct on the plasma membrane. The enhanced cyan fluorescent protein (ECFP) is used for the visualization and SNAP for the permanent binding of the SNAP-tag on the dimerizer. By recruiting an iSH2-mRFP-FKBP12 fusion protein to the cell membrane phosphatidylinositol 3-kinase (PI3K) is activated and PIP3 released. The inter-Src homology 2 domain (iSH2) is activating PI3K, red fluorescent protein (mRFP) is added for visualization and the FKBP12 mutant for Shld binding. PIP3 level is observed by a second messenger, the pleckstrin homology domain of Akt (PH) fused to green fluorescent protein (EGFP) (Feng et al., 2014). Additionally, PIP2 conversion to PI (4)P was made observable by Schifferer et al. by using Lck-ECFP-SNAP with a mRFP-FKBP-5Ptase and EGFP-PLCδ-PH fusion protein. PLCδ-PH is binding to PIP2 on the membrane and the 5Ptase is transforming PIP2 to PI (4)P. Thus, PIP2 levels on the membrane can be visualized by monitoring the release of EGFP-PLCδ-PH from the membrane (Schifferer et al., 2015).

There is always a high demand for tools to mimic or insert posttranslational modifications (PTMs). With the CID systems it is possible to address several PTMs, for instance, sumoylation (Zhu et al., 2006; Zimnik et al., 2009) and protein autoregulation like intein splicing (Mootz et al., 2003). Karginov et al. were able to allosterically activate kinases with CIDs to regulate phosphorylation levels. The fusion protein FAK-iFKBP did only show kinase activity by binding of Rapamycin together with the FRB protein. FAK is the focal adhesion kinase, whereas iFKBP is a truncated version of FKBP12 inserted into the kinase. The iFKBP insertion renders the kinase inactive. However, binding of Rapamycin restored kinase activity. Successful regulation at the same conserved site of the kinases Src and p38 shows the transferability of this system (Karginov et al., 2010). Additionally, kinase activation by the photoinducible Rapamycin derivative pRap was successfully tested (Karginov et al., 2011). Furthermore, another system for kinase control was established by Dagliyan et al. Fusion of a UniRapR-domain to a Src kinase exhibited regulatory activity by acting as a conformational switch. This UniRapR-domain simply consists of a FKBP12 and FRB fusion. While insertion of the UniRapR-domain at a specific site depleted Src activity completely, binding of Rapamycin or iRap fully restored the kinase activity. Comparing the effect of the Rapamycin activation of the Src-UniRapR fusion protein and an inactive Src mutant in single cells showed that the cells with the activated kinase started to spread and increase their cell area. Further studies of Src-UniRap activation in epidermal cells of zebrafish embryos showed a morphological change of the cell shapes resulting in less cell-cell contacts. This confirms the already known role of Src in tumor metastasis (Dagliyan et al., 2013).

Camacho-Soto et al. pursued another approach of CID directed kinase activation by creating ligand gated split kinases. They successfully split several kinases (Lyn, Fak, Src, and PKA) and fused their split domains with FKBP12 and FRB respectively. Addition of Rapamycin reconstitutes the enzyme and its activity (Camacho-Soto et al., 2014a). Further development also enabled split-phosphatases and moreover an orthogonal system of split-phosphatase and kinase (Camacho-Soto et al., 2014b). Here the orthogonality was obtained by using additional CIDs which utilize other ligand-receptor systems (abscisic acid).

A new area emerging in CID research are small-molecule protein degraders, the proteolysis targeting chimeras (PROTACs). Directed protein degradation with a built in on-switch can be a useful tool to discern a sudden knockout of a specified protein and the biological responses. PROTACs might be even used as new type of drug modality (Churcher, 2018). A PROTAC in general consists of a ligand binding to a ubiquitin ligase (E3) which is connected to a ligand binding the protein target. Polyubiquitination of proteins is known to be a marker for protein lifetime. By bringing the target protein in the proximity of the E3 ligase, its ubiquitination will be prolonged until the protein is recognized by the proteasome complex and degraded. The Crews group, which first reported the application of PROTACs, recently constructed FKBP12^F36V^ binding PROTACs to find new E3 ubiquitin ligases as PROTAC targets. To be able to assess the functionality of the PROTACs, a EGFP-FKBP12^F36V^ reporter system was applied. The EGFP degradation was a direct observable parameter for a positive outcome. Utilizing this fusion system theoretically renders any protein amenable to PROTAC induced degradation (Ottis et al., 2017). Recently, Nabet et al. constructed FKBP12^F36V^ degrading PROTACs containing the ligand dTAG51 or the ortho regioisomer dTAG13 (Figure 14), coupled to the cereblon E3-ligase ligand thalidomide.

Interestingly, the ortho regioisomer bound more selectively to the FKBP12 mutant than the original meta one. The dTAG system was made generalizable to study divergent proteins by introducing the CRISPR/Cas9 system to reach locus-specific insertion of FKBP12^F36V^. Treatment of cells with the fusion proteins by the selective dTAGs lead to rapid reduction in POI concentration. This was tested successfully for the bromodomain isoform BR4 and the oncoprotein KRAS^G12V^ in cells. The degradation of a luciferase coupled FKBP (luc-FKBP12^F36V^) was also tested *in vivo*. Mice were engrafted in their bone marrow with the human leukemia cell line MV4;11 carrying the luc-FKBP12^F36V^ vehicle. By administering the drug (dTAG) they were able to monitor a rapid and durable decrease in bioluminescense, which was reversible after withdrawal of the drug (Nabet et al., 2018). This is a promising start for future drug development built on PROTACs and a universal usable tool for protein degradation, although it has to be kept in mind, that the creation of fusion proteins always requires the test of functionality of the fusion protein. Furthermore, for the development of PROTAC-drugs a directly POI binding ligand has to be synthesized.

### Immunophilin Ligands Used in Reverse Dimerization

Interestingly, mutants for FKBP12 were also designed to reach dimerization without ligand treatment. These FKBP12^F36M^ mutants were created by Rollins et al. and could be disrupted by the addition of monomeric Shld1 derivatives like AP22542 and AP21998 (Rollins et al., 2000). With this reverse dimerization system Rivera et al. were able to regulate protein secretion through controlled protein aggregation in the ER. POIs were fused together with four equivalents of the FKBP12^F36M^ mutant and a signal sequence for ER transport. Additionally, a furin cleavage site was inserted between the FKBPs and the POI. The proteins carrying the signal sequence were assembled in the ER and started to build complexes, which were too big for vesicle transport. After ligand administration (AP22542 or AP21998, Figure 14) the constructs dissolved, and the POI hybrid was released. Finally, cleavage by furin unveiled its native form preventing a reaction of the immune system. This approach was already used to secret therapeutic proteins like proinsulin or the growth hormone (GH) in cells. The release of proinsulin was also tested *in vivo* with diabetic mouse models showing promising results (Rivera et al., 2000). Al-Bassam et al. used the same system to monitor movements of post-Golgi transport vesicles in neurons. Neuronal transmembrane proteins are known to be transported in vesicles, that traffic solely within the somatodendritic compartment to the dendrites. They created a pulse-chase system, which allows the spontaneous release of fluorescently labeled transmembrane proteins to follow the vesicles transport (Al-Bassam et al., 2012). Recently, the FKBP12^F36M^ mutant was improved by Barrero et al. who introduced a F36L mutation instead. This mutant dimerizes readily and has additionally better ligand binding capabilities and might lead to further utilization of this dimerizing system (Barrero et al., 2016). Tonthat et al. found a formation of dimers in the apo crystal structures of fungal FKBP12 (*A. fumigatus, C. albicans*, and *C. glabrata*). These dimers revealed the binding of a proline residue (located in the 80s loop of FKBP12) to the active site of another FKBP12 protein unit. A mono cysteine mutant (V91C) of *A. fumigatus* FKBP12 formed the dimer in solution by disulfide bridging. This might elucidate the substrate specifics of the PPIase site in FKBP12. Additionally, this putative FKBP12 self-catalysis might be a self-regulatory action of the protein (Tonthat et al., 2016).

## Conclusion

FKBP research has historically focused on human FKBP12, but more recently several other homologs have received increasing attention (e.g., FKBP12.6, FKBP51, AIPL1). Human FKBPs discussed in this review have been summarized in Table 1, to get a comprehensive idea of the key aspects of these proteins. Note that there are many more human FKBPs which are not mentioned due to poor characterization and/or understanding of their biophysical properties. Additionally, the effects of ligands on the respective proteins have been omitted since many effects are controversially discussed.

**Table 1:** Overview of the key aspects of human FKBPs discussed in this review.

**FKBPs**	**Gene**	**Domains**	**PPIase activity**	**Cellular location**	**Physiological distribution**	**Cellular role of FKBP**	**Crystal structures (human FKBPs)**	**Available ligands and binding affinity**
FKBP12	*FKBP1A*	FK	Yes	Cytoplasm	Expressed in most tissues, especially: Skeletal and cardiac muscle Central nervous system Peripheral nerves Blood cells	Regulation of RyR Regulation of TGFβ Muscle contraction in skeletal muscle	1A7X, 2PPN, 1FKJ and many more	Rapamycin (0.6 nM) FK506 (0.2 nM) FK1706 (0.5 nM) Ligand 4 (0.2 nM)
FKBP12.6	*FKBP1B*	FK	Yes	Cytoplasm	Skeletal and cardiac muscle	Regulation of RyR Cardiac muscle protection	41Q2, 41QC, 1C9H	Rapamycin (0.4 nM) FK506 (4.4 nM)
FKBP13	*FKBP2*	FK ER signal peptide ER retention sequence	Yes	Secretory pathway Endoplasm	Expressed in most tissues, especially: Pituitary gland Adenohypophysis	Regulator of protein homeostasis in long-lived plasma cells Protein folding Involved in AD	4NNR, 2PBC	Rapamycin (7.2 nM) FK506 (166 nM)
FKBP19	*FKBP11*	FK ER signal peptide	Yes	Secretory pathway Endoplasm	Expressed in most tissues, especially: Pancreas Duodenum	Role in collagen folding	Not available	No data available
FKBP22	*FKBP14*	FK ER signal peptide ER retention sequence EF-handmotif Ca^2+^ binding	Yes	Secretory pathway Endoplasm	Expressed in most tissues, especially: Tibia Intestine Stomach	Role in collagen folding	4MSP	No data available
FKPB65	*FKBP10*	FK (4x) ER signal peptide ER retention sequence EF-hand motif Ca^2+^ binding	Yes, unclear which domain (s)	Secretory pathway Endoplasm	Expressed in most tissues, especially: Pituitary gland Adenohypophysis	Role in collagen folding Modulates LH2 activity	Not available	No data available
FKBP25	*FKBP3*	FK	Yes	Nuclear	Skeletal muscle	Interaction with dsRNA Role in ischemia stress injury protection Microtubule-associated protein	1PBK, 5D75 and partial structures	Rapamycin (0.9 nM) FK506 (200 nM) BTHB domain binds RNA
AIPL1	*AIPL1*	FK TPR PRD	No	Nuclear	Retina Pituitary gland	Chaperone for PDE6 Mutations cause LCA	5U9A, 5U9I, 5U9J (only FKBP domain)	No data available
FKBP51	*FKBP5*	FK1 & FK2 TPR (3x)	Yes (only FK1 domain)	Cytoplasm	Highly abundant in most tissues	Negative regulation of glucocorticoid and progesterone receptors Involved in stress-related diseases, psychiatric disorders and obesity	1KT0, 5NJX, 5OMP and many more	Rapamycin (3.7 nM) FK506 (104 nM) SAFit1 (4 nM) SAFit2 (6 nM) SLF (6.3 nM)
FKBP52	*FKBP4*	FK1 & FK2 TPR (3x)	Yes (only FK1 domain)	Cytoplasm	Abundant in most tissues	Positive regulation of glucocorticoid and progesterone receptors	1P5Q and partial structures	Rapamycin (4.2 nM) FK506 (23 nM) FK1706 (14 nM)

Several themes that are noteworthy:

1. Besides the name, many FKBPs do not bind FK506 or do so poorly. They are better described as FKBP-like proteins. So far, the FK506-binding potential is closely correlated with sequence homology to the active site of FKBP12.
2. For pharmacological studies of FKBPs, FK506 or Rapamycin are suboptimal tools since they also block calcineurin or mTOR as confounding factors. Well characterized, highly potent, non-immunosupressive analogs are available such as FK1706 or bicyclic analogs. GPI1406 is an extremely poor FKBP ligand.
3. Many FKBPs do not have a measurable PPIase activity, although some can or might be activated by biochemical stimuli. For weak PPIase activity care has to be taken of avoiding contaminations with high PPIase activity (e.g., *E. coli* SlyD). Binding of ligands to FKBPs should always be confirmed by biophysical assays (fluorescence polarization, isothermal calorimetry, NMR), which are available for many FKBPs.
4. Even for FKBPs with a clearly established PPIase activity, the physiological role of this activity remains to be established. The biochemical functions documented so far invoke protein-protein interactions (e.g., FKBP12/12.6-RyR, FKBP12-TGFβR1, AIPL1-Farnesyl-PDE6).
5. The FK506-binding pocket of FKBPs can be plastic and this can be important for their function (AIPL1, FKBP51). It also offers new opportunities for the development of selective FKBP ligands.
6. Several FKBPs have intertwined functions with overlapping substrates (FKBP12/12.6-RyR1/2, FKBP51/52-GR/AR). It is essential, to perform control experiments and check for compensatory effects in genetic studies. Additionally, more selective pharmacological tools are required.

Finally, it should be kept in mind that there are still many (human) FKBPs that we hardly know anything about.

## Author Contributions

JMK primarily wrote sections FKBP12 and FKBP12.6, FKBP13, and other endoplasmic FKBPs, FKBP25, and Microbial FKBPs, AMV section Chemical tools/Chemically induced dimerization utilizing FKBPs, MB sections FKBP51, and FKBP52, and FH sections Introduction, AIPL1, and Conclusion. All authors read and approved the final version.

### Conflict of Interest Statement

The authors declare that the research was conducted in the absence of any commercial or financial relationships that could be construed as a potential conflict of interest.
